# Next generation genetic screens in kinetoplastids

**DOI:** 10.1093/nar/gkaf515

**Published:** 2025-06-18

**Authors:** James Budzak, T Nicolai Siegel

**Affiliations:** Division of Experimental Parasitology, Faculty of Veterinary Medicine, Ludwig-Maximilians-Universität München, Munich, 82512, Germany; Biomedical Center, Division of Physiological Chemistry, Faculty of Medicine, Ludwig-Maximilians-Universität München, Munich, 82512, Germany; Division of Experimental Parasitology, Faculty of Veterinary Medicine, Ludwig-Maximilians-Universität München, Munich, 82512, Germany; Biomedical Center, Division of Physiological Chemistry, Faculty of Medicine, Ludwig-Maximilians-Universität München, Munich, 82512, Germany

## Abstract

The genomes of all organisms encode diverse functional elements, including thousands of genes and essential noncoding regions for gene regulation and genome organization. Systematic perturbation of these elements is crucial to understanding their roles and how their disruption impacts cellular function. Genetic perturbation approaches, which disrupt gene expression or function, provide valuable insights by linking genetic changes to observable phenotypes. However, perturbing individual genomic elements one at a time is impractical. Genetic screens overcome this limitation by enabling the simultaneous perturbation of numerous genomic elements within a single experiment. Traditionally, these screens relied on simple, high-throughput readouts such as cell fitness, differentiation, or one-dimensional fluorescence. However, recent advancements have introduced powerful technologies that combine genetic screens with image-based and single-cell sequencing readouts, allowing researchers to study how perturbations affect complex cellular phenotypes on a genome-wide scale. These innovations, alongside the development of CRISPR–Cas technologies, have significantly enhanced the precision, efficiency, and scalability of genetic screening approaches. In this review, we discuss the genetic screens performed in kinetoplastid parasites to date, emphasizing their application to both coding and noncoding regions of the genome. Furthermore, we explore how integrating image-based and single-cell sequencing technologies with genetic screens holds the potential to deliver unprecedented insights into cellular function and regulatory mechanisms.

## Graphical Abstract

**Graphical Abstract:**
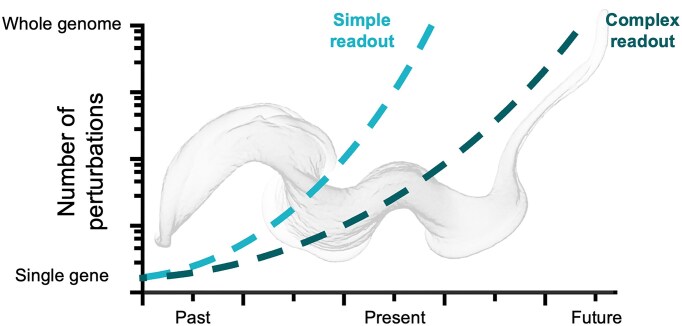

## Introduction

Deciphering the function of genes and genetic regulatory elements is critical for understanding how cells work. One of the primary means to determine genome function is through perturbation. Over the past decade, tools to carry genetic perturbations have increased significantly in many organisms and can now be scaled to the order of thousands of genes and even genome wide. These “genetic screens” offer a means to identify gene function at a massive scale in an unbiased manner and have been driven in part by the discovery and development of CRISPR–Cas9 [1].

Here, we focus on the current and potential future applications of genetic screens to study kinetoplastids. Within the kinetoplastids several organisms cause deadly diseases in humans, including *Leishmania* species, *Trypanosoma brucei*, and *Trypanosoma cruzi*. These parasites represent a group of eukaryotes that is evolutionarily highly divergent from better studied organisms such as yeast, *Caenorhabditis elegans* or Drosophila and that have compact predominantly diploid genomes of 30–60 Mb with between 8,000 and 10,000 protein coding genes. Almost all protein coding genes are transcribed from polycistronic transcription units (PTUs), which contain many, often >100, functionally unrelated genes transcribed from a single transcription start site and then processed into mature mRNA by trans-splicing [2]. As such, there is little transcriptional control in these organisms and mRNA levels are predominantly regulated post-transcriptionally by RNA-binding proteins. In addition, kinetoplastid parasites lack canonical nonhomologous end joining (NHEJ) pathways and instead primarily rely on homologous recombination (HR) for the repair of double strand breaks (DSBs), an important biological consideration for the design of genetic screens (discussed in detail below) [3]. Over the years, kinetoplastids have been used to study a myriad of unique and evolutionarily conserved biological processes. These include antigenic variation [4], quorum sensing [5], cell motility [6], transcription of protein coding genes by RNA polymerase I [7], trans-splicing [8], polycistronic transcription [2], and genetic code reassignment [9]. Importantly, this group of organisms has high genetic tractability, allowing them to be used as model systems to study these different processes.

**Figure 1.**
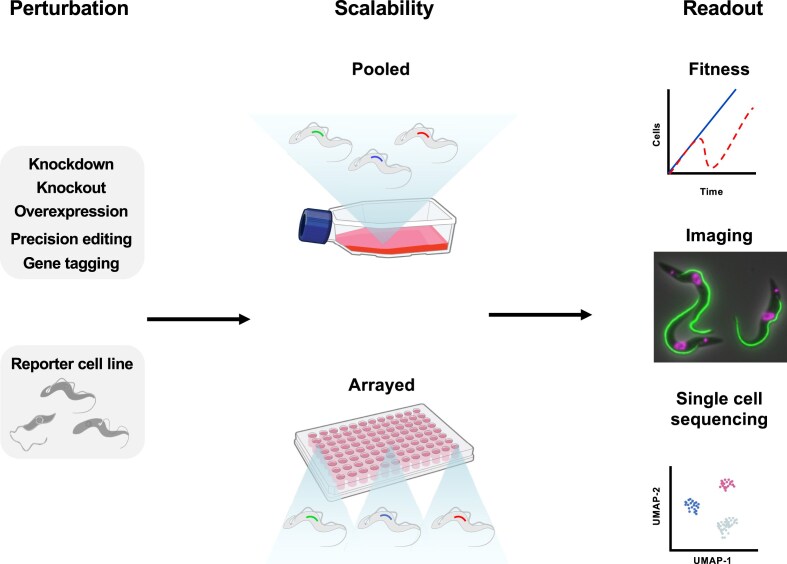
Genetic screens require a specific perturbation, scalability, and a readout. Each screen starts with specific DNA sequences encoding for specific perturbations, either a knockdown, knockout, overexpression, precision edit or gene tagging which are transfected into a cell line of interest. This transfection can either be in individual wells or flasks (arrayed) or into a single flask of cells (pooled). After incubating cells or inducing the perturbation, the effect of the perturbations is determined by a readout which can be cell fitness, imaging, or single-cell sequencing. The microscopy image shown is for PFR2 (Tb927.8.4990) and was acquired from TrypTag. The schematics used for the parasites in this and all subsequent figures were adapted from [189]. The illustrations for the flask and 96-well plate were sourced from the NIAID NIH BIOART. Source: bioart.niaid.nih.gov/bioart/303 and bioart.niaid.nih.gov/bioart/7, respectively.

**Figure 2.**
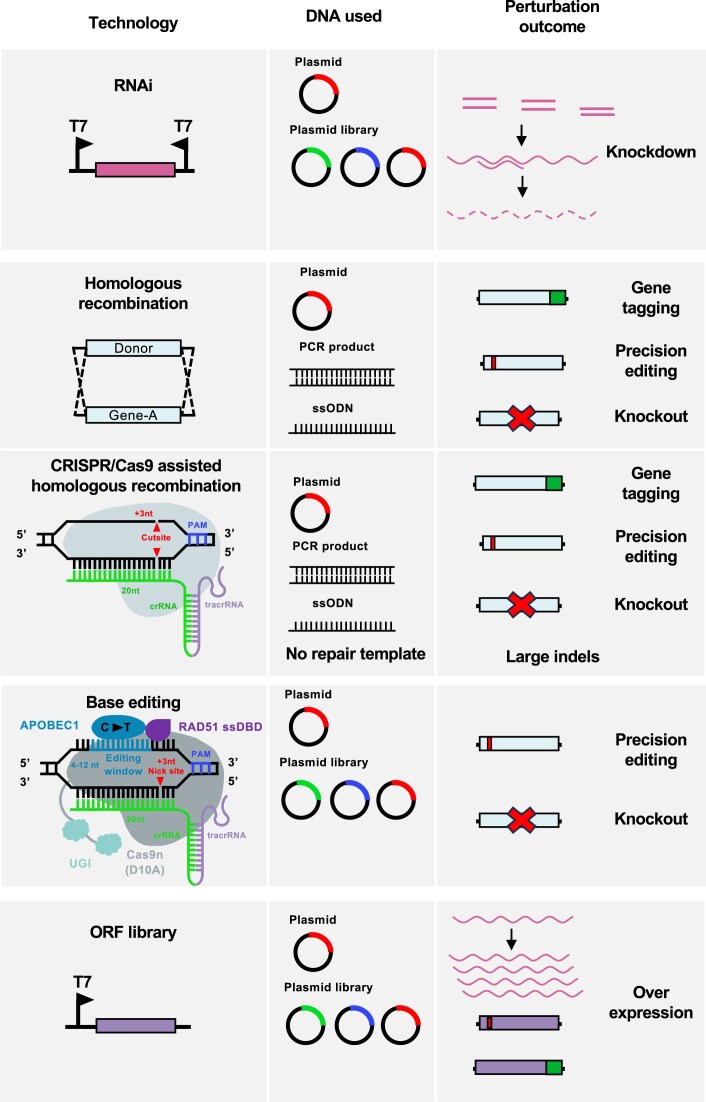
Overview of different perturbation strategies used in kinetoplastids for genetic screens. Schematics showing different perturbation technologies used in kinetoplastids. The type of DNA (plasmid, plasmid library, PCR product, and ssODN) used with each technology is shown as is the expected perturbation outcome, either a knockdown, knockout, overexpression, precision editing, or gene tagging.

**Figure 3.**
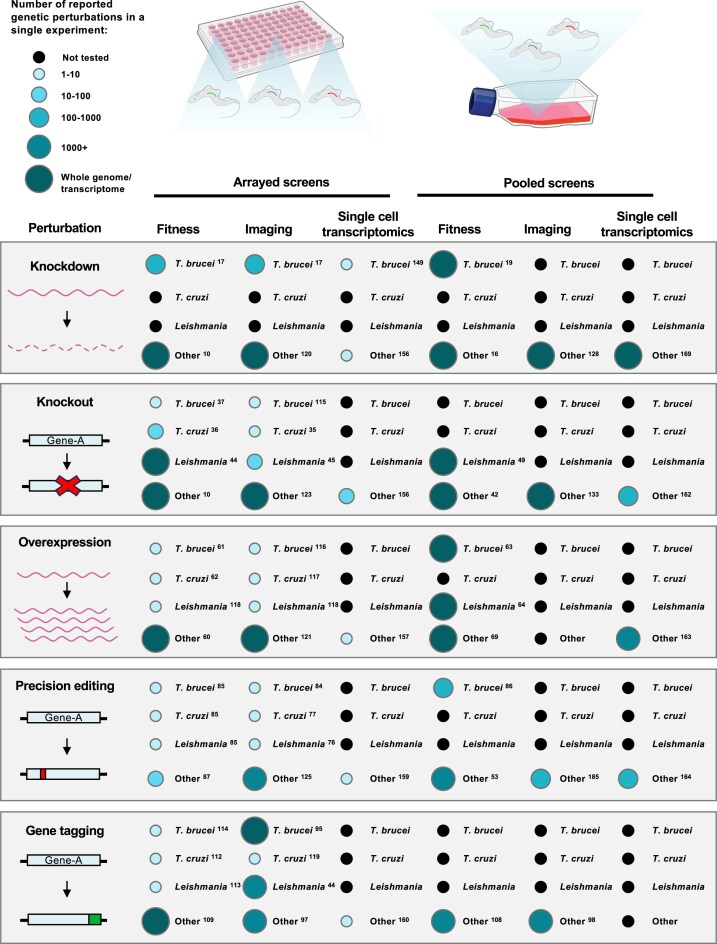
Overview of scale of genetic screens performed in kinetoplastids and other organisms. Comparative chart showing the number of genetic perturbations performed in a single experiment for knockdown, knockout, overexpression, precision editing, or gene tagging using either arrayed or pooled genetic screens. The size and color of the circles indicates the number of reported perturbations in a single experiment for the respective organism. “Other” refers to mammalian cells, yeast, or Drosophila. In instances where multiple publications have reported screens within the same size range, only one example is cited. Superscript numbers refer to the cited publication found in the references.

Genetic screens require three key features: a specific perturbation system (most commonly a knockout, knockdown, overexpression, precision edit, or gene tagging; Figs 1 and 2), the ability to be scalable and a clear readout allowing interpretation of the experimental question being addressed. Genetic screens can be performed either as an array or as a pool. In an arrayed screen, specific genetic perturbations are generated in separate wells or flasks and individual perturbations are analysed one at a time [3, 10]. In contrast, in a pooled screen, multiple genetic perturbations are generated and assessed simultaneously in a single flask (Fig. 1). Pooled screening offers several advantages over arrayed screening in that it is scalable, less labour-intensive and generally of higher throughput, allowing whole genome screens to be carried out with relative ease (Fig. 3). Typically, arrayed screening is used in instances where homology repair templates must be used for gene perturbation, such as with knockout or precision editing screens (Table 1). Pooled screening, on the other hand, is used when no repair template is required for perturbation, such as with knockdown mediated by RNA interference (RNAi) and overexpression screens (Table 1). Perturbation methods which require DSBs at the target gene typically require a repair template to ensure precise modification, which in turn affects scalability.

**Table 1.** Overview of which perturbation strategies can be used for arrayed or pooled whole genome screens and whether they have been performed in kinetoplastids. The schematics used for the parasites in this table were adapted from [189]

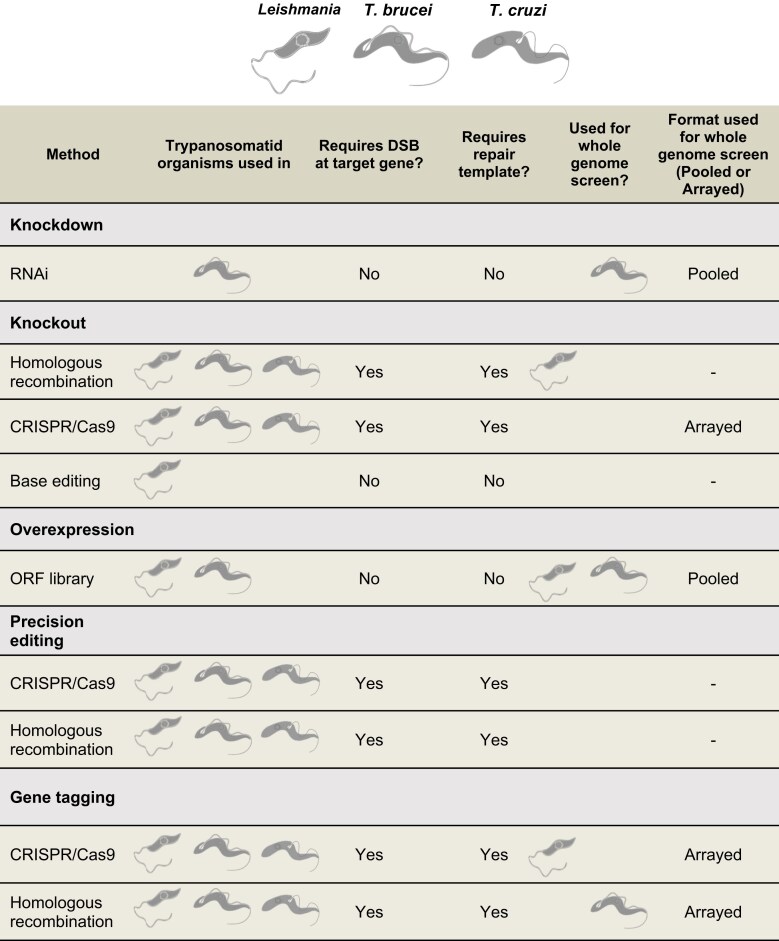

In this review, we discuss recent developments and potential future applications of genetic screens in kinetoplastid parasites. In addition, we discuss new tools which allow complex readouts of genetic screens. Almost all genetic screens performed to date in kinetoplastids have used cell fitness as a readout. However, several technologies which allow image-based and single-cell transcriptome-based readouts of pooled genetic screens have been developed in the last few years.

These new types of genetic screening approaches will allow researchers to address biological questions in kinetoplastids that have not been possible before. For instance, in bloodstream form *T. brucei*, we have previously shown that the major surface antigen gene forms a stable *trans* interaction with a major splicing locus in the nucleus [11, 12]. However, it is still not fully understood what factors are needed to maintain the interaction between these two genomic loci. Using pooled image-based genetic screens, it would be possible to identify factors that regulate the localization of these two sub-nuclear compartments. Indeed, such screening approaches could also be used to identify factors important for other aspects of cell morphology, protein localization or cell-to-cell interactions. In addition, in kinetoplastids our understanding of gene function is still extremely limited, with functional studies only applied in detail to a small subset of genes. By combining pooled genetic screens with single-cell RNA-sequencing readouts, it would be possible to obtain transcriptomic profiles after perturbation for hundreds or thousands of genes and thus improve our understanding of gene function at an unprecedented scale.

Together, these advancements can now close the gap on genotype-to-phenotype relationships at a genome wide scale and have the potential to further unravel evolutionarily conserved and parasite-specific biological processes in this fascinating group of organisms.

## Genetic screens using knockdown

Gene knockdowns were first described in *T. brucei* over 25 years ago [6, 13]. Since then, RNAi has become a staple tool to investigate gene function. Although RNAi is not present in *T. cruzi* or *Leishmania* species, hundreds of individual genes have been knocked down using RNAi in *T. brucei*. However, gene-by-gene knockdown is time consuming and is not readily amenable to whole genome screens. The ability to perform whole genome RNAi screens in *T. brucei* was facilitated by the development of the first RNAi plasmid library. This was generated by shearing *T. brucei* genomic DNA followed by cloning into a plasmid in between opposing tetracycline-inducible T7 RNA polymerase promoters. Importantly, the previously developed tetracycline-inducible system was essential to be able to control the timing of double-stranded RNA (dsRNA) production [14]. This allowed RNAi screens to be performed in a pooled format, increasing the throughput by several orders of magnitude which resulted in *T. brucei* being the first organism ever in which a whole genome RNAi screen was performed [15, 16]. Prior to this, the largest RNAi screen in *T. brucei* was a gene-by-gene knockdown of 210 genes located on chromosome I [17].

Since then, RNAi screens in *T. brucei* have been developed further (by increasing transfection efficiency using I-SceI to introduce DSBs [18]) and have been used extensively to elucidate various biological questions (∼68 screens published to date). The development of RNAi target sequencing (RIT-seq) in *T. brucei* in both life cycle stages has provided loss-of-function information for all genes in the genome (Fig. 3) [19]. RNAi library screening has been used to study mechanisms of drug action/resistance [20, 21], identify regulators of gene expression [22], identify cell cycle regulators [23], and elucidate signalling pathways [24] (Table 2) (for a comprehensive overview of RNAi screens in *T. brucei* see [25]). Due to RNAi library screens being highly effective in *T. brucei*, the need to generate CRISPR–Cas9 technologies for large scale knockout screens has been less pertinent. Particularly as a repair template is required for conventional CRISPR–Cas9-mediated knockout in kinetoplastids meaning pooled genetic screens are not immediately possible (discussed later). In addition, other kinetoplastid parasites such as *Leishmania* and *T. cruzi* do not have functional RNAi pathways [26]. This has precluded the use of large-scale genetic screens in these organisms.

**Table 2.** Overview of genetic screens performed in kinetoplastid parasites. *Number as of April 2024, project is ongoing. ^‡^ RNAi fragments detectable for 7435 genes after library transfection

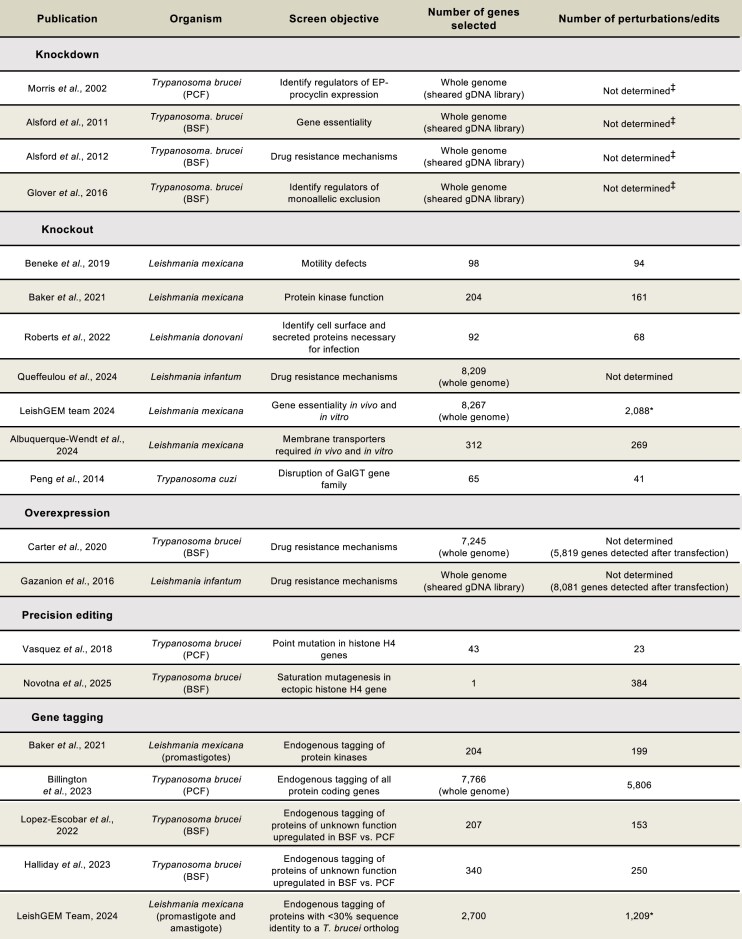

Cas13 is another CRISPR system which has gained considerable attention in recent years. Unlike Cas9–Cas12, Cas13 is a programmable RNA-guided RNA targeting endonuclease [27]. Cas13 has been used in several organisms for both target gene knockdown and overexpression including for large scale pooled genetic screens [28]. In addition, because Cas13 (like Cas12) has intrinsic CRISPR RNA (crRNA) processing activity, multiplexed gene perturbation is possible using this enzyme [28]. However, currently no Cas13 system has been described in any protozoan parasites although this system has several potential applications for pooled genetic screens in this group of organisms (discussed later).

## Genetic screens using knockout

For many years, genetic knockouts in kinetoplastids were generated by transfecting cells with plasmids containing drug selection markers to replace genes of interest by HR (Fig. 2) [29]. Cre/loxP has also been used to perform gene knockouts, including conditional gene knockouts, removal of drug resistance genes, and near scarless genetic modification [30, 31]. Since the discovery of CRISPR–Cas9, genetic knockouts using this system have been adapted to kinetoplastids using inducible, constitutively expressed or recombinant Cas9 [32–37] for both small and large scale genetic screens. CRISPR–Cas9-mediated knockouts have several benefits over plasmid-based and Cre/loxP-based knockouts, in that they are more efficient, can achieve deletion of multiple alleles in a single experiment, and can be performed using PCR-generated repair templates [38, 39]. In general, the use of an inducible CRISPR–Cas9 system for genetic knockouts is preferable as it allows the timing of knockouts to be precisely controlled. This permits the study of essential genes, minimizes potential off-target and secondary effects, and allows the effects of gene knockouts to be studied after the introduction of a particular stimulus or challenge to the cells. For simpler analyses (such as whether a gene merely affects fitness or not), inducible systems are not necessary. Indeed, these same caveats also apply to the use of inducible systems for other types of genetic screens. In *T. brucei*, tetracycline-inducible systems are routinely used for CRISPR–Cas9 expression [33]. In *Leishmania* and *T. cruzi*, no CRISPR–Cas9-inducible systems have been reported. However, in *Leishmania*-inducible knockouts can be performed using the DiCre system, which relies on chemically induced dimerization of the Cre recombinase after the addition of rapamycin [40]. As discussed below, a split Cas9 variant that can be dimerized into an active state upon addition of rapamycin has been used successfully in *Toxoplasma gondii* [41] and may be applicable to inducible CRISPR–Cas9 systems in kinetoplastids.

Typically, to perform pooled genetic knockout screens using CRISPR–Cas9, a canonical NHEJ mechanism is needed in the target cells. In NHEJ competent cell types, DSBs generated by CRISPR–Cas9 will generate small insertions or deletions (indels) resulting in a loss-of-function in the target gene which does not require a repair template. In contrast, in cell types which predominantly use HR to repair DSBs, a repair template is required to facilitate efficient disruption of the target gene. Therefore, organisms which primarily use NHEJ to repair DSBs are amenable to pooled CRISPR–Cas9 knock out screens, while organisms which use HR to repair DSBs are only amenable to arrayed CRISPR–Cas9 knockout screens. The use of a NHEJ repair pathway has been exploited for whole genome pooled CRISPR–Cas9 knockout screens in mammalian cells [42] and *T. gondii* [43]. However, many kinetoplastid parasites predominantly use HR mediated DSB repair, meaning pooled CRISPR–Cas9 screening is not immediately possible.

Nevertheless, several large-scale arrayed genetic screens using CRISPR–Cas9-mediated knockout have recently been performed in *Leishmania* (Table 2). One of the most ambitious of these is a project currently underway called LeishGEM that aims to carry out a genome wide deletion screen using CRISPR–Cas9 in *Leishmania mexicana* [44]. This knockout screen uses the approach described in [38], in which target genes are cut by Cas9 and repair templates are generated by PCR amplification of drug selection makers with 30 nt homology arms. Similarly to other genome wide functional analyses, this resource will be of immense value in elucidating the function of all genes in the *Leishmania* genome.

Knockout screens targeting functionally related groups of proteins have also been performed in *Leishmania* using CRISPR–Cas9. Such screens have been used to identify proteins critical for motility and sandfly infections [45–47], kinases involved in differentiation [46], and transporter proteins required for macrophage and mice infections [48]. In these screens, generation of knockout mutants was performed in an arrayed format, followed by pooling of the different knockout cell lines for subsequent functional analyses. In addition, a genome wide pooled CRISPR–Cas9 screen was recently reported in *L. infantum* to investigate anti-leishmanial drug resistance [49]. However, gene deletions were performed in the absence of any repair template. This is problematic, as it has previously been shown in *Leishmania* that in the absence of a repair template, CRISPR–Cas9-induced DSBs can trigger deletions of ∼70 kb with co-deletion of multiple genes [50]. The precision, specificity, and interpretability of such screens is therefore unclear.

Another recent study published in *Leishmania* reported a more robust strategy for performing pooled genetic knockouts without the need for repair templates by base editing using a cytosine base editor (CBE). CBEs contain an engineered deaminase domain fused to a variant of Cas9, typically a Cas9 nickase (nCas9) which harbours a D10A mutation which results in nicking of DNA rather than a DSB [51]. Targeting of nCas9 to a gene of interest via a single guide RNA (sgRNA) allows the fused deaminase domain to catalyse the deamination of cytidine to uridine within an “editing window” typically +4 to +8 nt upstream of the PAM [51]. This uridine is then converted to thymidine during DNA replication resulting in C-to-T point mutations. This approach (termed CRIPSR-STOP) allows the introduction of premature stop codons in target genes using only a sgRNA with no repair template or DSB induction [52]. CBEs have been extensively optimized in mammalian cells, with several engineered variants displaying exceptional specificity and editing efficiency which have been used for a growing number of large scale pooled loss-of-function screens [51–53].

A recent paper has now described the development and optimization of the CBE variant hyBE4max for CRISPR-STOP in several *Leishmania* species [54]. hyBE4max is a fusion protein composed of an APOBEC-1 deaminase domain, a single-stranded DNA-binding domain (ssDBD) from RAD51, nCas9, and a uracil glycosylase inhibitor (UGI) domain (Fig. 2) [55]. Addition of the RAD51 ssDBD between nCas9 and the APOBEC-1 deaminase extends the CBE editing window to 4–12 nt upstream of the PAM [55], while addition of the UGI domains prevents base excision repair mediated reconversion of uridine to cytidine [56]. As a proof-of-concept, the authors were able to induce premature stop codons via expression of a hyBE4max fusion protein and sgRNA from a single plasmid with exceptional editing efficiencies (up to 100%) in both ectopic, endogenous, and multi-copy genes [54].

However, several important considerations are worth noting. First, potential off-target mutations induced by this CBE were not tested. Second, introduction of premature stop codons results in degradation of mRNA by nonsense-mediated decay (NMD) in mammalian cells [57]. However, a canonical NMD pathway is not thought to operate in several kinetoplastids [2, 58]. Therefore, premature stop codons may not fully ablate target mRNA levels and instead result in the expression of truncation mutants which may still possess partial or full activity. Lastly, in this study sgRNA expression was driven via an episome. In general, pooled genetic screens are performed with stably integrated perturbation constructs that integrate into a single copy locus to prevent multi-gene perturbations, mitigate the effects of episomal copy number, and increase transfection efficiency. In relation to this latter point, a follow-up study showed that AsCas12a ultra can be used to induce a DSB in a single copy locus to facilitate highly efficient integration of CBE sgRNAs in several *Leishmania* species [59], with similar efficiencies to that seen with I-SceI mediated DSB induction for transfecting whole genome RNAi libraries in *T. brucei* [18].

Combined, these two studies [54, 59] provide a significant technological advancement for conducting large scale and whole genome pooled genetic screens in *Leishmania* parasites (and potentially other kinetoplastid parasites).

## Genetic screens using overexpression

Aside from loss-of-function knockout and knockdown genetic screens, gain-of-function genetic screens mediated by protein overexpression can be used to determine gene function [60]. Overexpression of genes in kinetoplastids is typically achieved by inserting an ectopic copy of the gene of interest in the genome followed by expression using a strong promoter (i.e. T7 or rDNA) which may be inducible (Fig. 2) [61, 62]. Whole genome pooled overexpression screens have been performed in both *Leishmania* and *T. brucei* to study drug resistance mechanisms [63–66]. These overexpression libraries were generated by randomly sheared genomic DNA fragments, or, by individual PCR amplification of all protein coding genes followed by cloning into an overexpression plasmid. Overexpression libraries have also been used in *T. brucei* to identify proteins which either stabilize or destabilize mRNA molecules when tethered to their 3′UTR [67]. However, overexpression libraries that incorporate randomly sheared genomic DNA fragments are limited by insert size, cannot be used for targeted overexpression libraries and lack precise expression of defined ORFs. Furthermore, generation of ORF libraries by PCR amplification of all genes in an arrayed format is very laborious.

CRISPR interference/activation (CRISPRi/a) is another method for performing loss-of-function and gain-of-function genetic screens by recruiting dCas9 fused to an epigenetic regulator to target gene promoters to inhibit or enhance transcription. CRISPRi/a has been used for large scale and whole genome genetic screens in mammalian cells to investigate mechanisms of gene expression and drug resistance [68, 69].

However, pooled CRISPRi/a screening is not possible in kinetoplastid parasites as many genes are transcribed from PTUs with a single promoter and regulation occurs predominantly at the post-transcriptional level [2, 70–73]. Intriguingly, this means that CRISPR–Cas technologies which target RNA such as Cas13 could potentially be used to upregulate/downregulate genes via fusion of dCas13 to regulators of translation. This approach has recently been shown to work in mammalian cells [74] and bacteria [75], by using sgRNAs against the 5′UTRs of target genes which recruit dCas13 proteins fused to regulators of translation. As all genes are constitutively expressed in kinetoplastids, whole genome sgRNA libraries could be designed to recruit dCas13 fusion proteins to UTRs of target genes to enhance or supress translation.

## Genetic screens using precision editing

Precision editing allows the introduction of specific point mutations or precise tagging or cutting of target genes. The effect of individual point mutations on gene function can be investigated by ectopic expression of a mutagenized copy [76, 77], although this approach is not readily scalable to large-scale genetic screens. Large-scale mutagenesis screens can be performed using chemical methods or transposons, which randomly introduce point mutations or indels across the genome, respectively [78, 79]. For more precise screening of mutant populations, multiplexed assays of variant effects (MAVEs) can be used, which allow the generation and analysis of many thousands of genetic variants or mutants in parallel. Within MAVEs, different types of assays can be used to study coding or regulatory elements. For instance, to study protein variants, deep mutational scanning (DMS) can be used to generate libraries of protein coding sequences containing one or many specific amino acid substitutions. DMS has recently been used in *T. brucei* to study RBP6 which is a master regulator of procyclic-to-metacyclic differentiation [80]. Here, the authors identified key amino acids and domains in RBP6 that are required for this differentiation process [81]. In another study in *T. brucei*, DMS was used to identify fitness conferring amino acids in the RNA editing protein KREPB4 [82]. In both cases, the mutagenized protein coding sequences were generated by error prone PCR followed by cloning mutagenized protein sequences into plasmid libraries. In addition to DMS, massively parallel reporter assays (MPRAs) are another high-throughput screening approach to determine the activities of up to many thousands of regulatory genetic sequences simultaneously. MPRAs have recently been used in *T. brucei* to identify sequences that control mRNA stability by fusing a whole genome plasmid library to the 3′ end of a tuneable negative/positive selection marker, allowing the identification of thousands of regulatory 3′UTR sequences [83]. Together, these approaches offer an extremely powerful means to assess many thousands of sequence variants at once. One caveat, however, is that they do not allow targeted mutagenesis at defined positions within endogenous genes of interest.

The ability to carry out precise edits on endogenous genes has been greatly facilitated by the development of CRISPR–Cas9. In *T. brucei*, Cas9 has been used to edit the histone H4 gene family to introduce point mutations that mimic a constitutively nonacetylated state. This facilitated the incorporation of point mutations with no drug selection marker in ∼90% of this ∼43 copy gene family after continuous editing, a feat that would be near unattainable in the pre-CRISPR era [34]. In addition to introducing point mutations, CRISPR–Cas9 has also been used for precision tagging of genes in *T. brucei* without the need for additional insertion of drug selection markers or modification of endogenous UTRs [84].

Aside from CRISPR–Cas9, it was recently shown that oligo targeting could be used for efficient, marker free and multi-base editing in *T. brucei*, *L. donovani*, and *T. cruzi* [85]. Here, the authors transfected cells with short (51 bp) single-stranded oligodeoxynucleotides (ssODNs) to introduce mutations into genes involved in drug sensitivity. This approach was used to perform saturation mutagenesis at a single amino acid position in the CRK12 gene to identify drug resistant conferring mutations. A similar approach was also used in a recent pre-print to perform saturation mutagenesis of 6 lysine residues in histone H4 [86]. Here, the authors deleted the entire histone H4 array (consisting of 43 genes in a 15-kb array) and inserted a single ectopic histone H4 gene under the control of a T7 promoter. This ectopic histone H4 gene was then edited using 384 ssODN repair templates containing different amino acid substitutions. However, unlike the previous study, here CRISPR–Cas9 was used to introduce DSBs in the ectopic histone H4 gene to increase the efficiency of histone H4 editing.

Lastly, as mentioned earlier, CRISPR–Cas9-mediated base editing can be used to introduce specific point mutations in genes of interest to cause a genetic loss-of-function. However, base editing can also be used to introduce a wider range of mutations, particularly when combing cytidine base editors with adenine base editors [87]. In human cells, base editing has been used to generate over 52 000 individual mutants in a single pooled genetic screen to investigate drug resistance mechanisms [53].

## Genetic screens using gene tagging

Determination of protein localization is a fundamental tool to understand cellular function. For many years, endogenous tagging of genes with fluorescent proteins has been used to uncover protein function and discover previously unidentifiable cellular structures. A seminal example of this comes from Akiyoshi and Gull [88]. Prior to this study, no proteins of the kinetochore had been identified in any kinetoplastid parasites due to high sequence divergence from kinetochores in other organisms. The authors carried out a screen using plasmid-based epitope tagging [89] to tag a cohort of 28 proteins which were upregulated later during the cell cycle. Using this approach, a single protein with a characteristic kinetochore localization (KKT1) was identified. Subsequent chromatin immunoprecipitation and DNA–FISH experiments confirmed KKT1 was associated with centromeres while reciprocal IPs identified a further 18 proteins forming the kinetoplastid kinetochore (Table 2). This study highlights the power of how protein localization can be used to discover and illuminate novel cell biology. Endogenous tagging using plasmid-based approaches has also been used to generate localization maps for functionally related classes of proteins such as the nuclear pore and chromatin associated proteins in bloodstream form (BSF) *T. brucei* [90, 91].

Endogenous tagging of proteins using homologous recombination with plasmid-based repair templates although efficient is not easily scalable and cannot be readily done in a high-throughput manner. In recent years, the development of high-throughput tagging techniques has led to significant advances in the ability to tag hundreds to thousands of genes (Table 2). In *Trypanosoma* and *Leishmania*, toolkits have been developed which allow endogenous tagging of genes using PCR generated repair templates [38, 39, 92]. Recent updates to these toolkits now allow tagging with a wider range of fluorescent proteins and epitope tags [93] as well as tagging without modifying endogenous UTR’s through the use of 2A peptides [94].

These breakthroughs have facilitated genome wide protein localization maps in procyclic form *T. brucei* through the TrypTag project, which used PCR-generated repair templates to tag 7,766 proteins with mNeongreen (mNG) [92, 95]. This is significant, considering that endogenous tagging of all protein coding genes has only been achieved in one other eukaryotic organism (*Saccharomyces cerevisiae*) [96] (Fig. 3). In human cell lines, CRISPR–Cas9 has been used to endogenously tag thousands of genes [97, 98]; however, this has not been conducted at a genome wide scale. The impact of TrypTag is still in its infancy; however, several studies using this protein localization atlas have already demonstrated its power to discover novel cell biology in both *T. brucei* life cycle stages. Examples of this include a complete proteome of the mitochondria [99], identification of nuclear bodies important for antigen expression [12], biochemical properties of nucleolar targeting [100], and identification of factors important for ciliogenesis [101]. For a comprehensive overview of the current impact of TrypTag see [102].

These genome wide localization maps for procyclic form *T. brucei* and yeast were made possible owing to high levels of efficient HR in these organisms, allowing transfection of PCR-generated repair templates with short homology arms [95, 96]. In many other organisms (and even life cycle stages), rates of HR are not efficient enough to facilitate such an approach, requiring individual cloning of longer homology arms into plasmids to serve as repair templates. However, CRISPR–Cas9-based gene editing has changed this, allowing tagging of genes with repair templates containing homology arms as short as 30 nt which can be generated by PCR in high-throughput arrayed formats.

In bloodstream form *T. brucei*, CRISPR–Cas9 has been used to determine the localizations of proteins upregulated in the mammalian infective stage compared to the insect stage of the parasite [103]. Here, the authors successfully tagged 153 proteins with mNG using ∼30–80 nt homology arms and were able to identify the first protein specifically localized to the Expression Site Body (called ESB1). The ESB is a nuclear body which is essential for monogenic expression of the Variant Surface Glycoprotein (VSG) antigen surface coat. This study is significant as although the ESB was discovered over 20 years prior to this [104], no protein specific to this structure had been identified [105]. Further characterization of this protein identified through high-throughput tagging will likely further reveal mechanisms of monoallelic exclusion in *T. brucei*. A follow up of this study using the same CRISPR–Cas9 tagging approach included additional localizations for 250 proteins in BSF cells, providing further insight into stage specific adaptations in this parasite [106].

CRISPR–Cas9-based high-throughput tagging by transfecting PCR-generated repair templates has also been conducted in *L. mexicana*. In one such study, Baker *et al.* selected 206 *Leishmania* protein kinases for endogenous tagging with mNG using 30 nt homology arms. The authors successfully localized 197 proteins, showing that protein kinases localize to a diverse range of cellular compartments, highlighting kinases which may have organelle specific functions [46].

A large scale tagging project called LeishTag (as part of the LeishGEM project) is currently underway in *L. mexicana* [44]. This approach aims to endogenously tag 2700 proteins with mNG using CRISPR–Cas9 by arrayed transfections with PCR generated repair templates and sgRNAs. As *L. mexicana* proteins share a high degree of sequence identity with *T. brucei* proteins (for which the TrypTag project has already provided localization data), only proteins with <30% sequence identity in *L. mexicana* will be tagged. Importantly, this resource will contain localization data for two different life cycle stages (promastigotes and axenic amastigotes).

In addition to using high-throughput tagging for determining protein localization, high-throughput tagging can also be used to insert functional tags on proteins for genetic screening purposes. In mammalian cells and yeast, high-throughput tagging experiments have been used to carry out screens for: protein degradation effectors using HaloTags [107], gene regulators using TetR/λ-N tags [108], and for genetic knockdowns using degron tags [109].

In *T. gondii*, Smith *et al.*, recently, described a high-throughput endogenous tagging approach using CRISPR–Cas9 [110] to tag genes with mNG and a minimal auxin-inducible degron (mAID) which allows both protein localization and inducible protein degradation. Importantly, this strategy is compatible with cloning tagging plasmids as libraries which can be transfected as pools. The resulting population contains a collection of cells each with a single protein tagged which can then be cloned out and sequenced to study individual proteins. Using this approach, the authors selected 147 protein kinases for tagging. Similarly, in *Plasmodium* a small screen used knock sideways to tag 27 proteins with both a fluorescent protein and a FKBP domain which can be used to inducibly re-localize proteins to an ectopic cellular compartment to ablate their function [111]. Tagging proteins with destabilization domains has also been used in *T. cruzi* and *L. major* for selective protein knockdown [112, 113], while in *T. brucei* the AID system has recently been shown to be effective at inducible protein degradation [114]. Currently, no such degradation system has been used for a large-scale genetic screen in any kinetoplastid parasite.

In summary, high-throughput tagging can be used to generate localization maps of proteins and generate libraries of tagged proteins for both functional and perturbation screening purposes thus providing valuable resources to dissect gene function.

## Pooled genetic screens with image-based profiling

Assessing phenotypic changes by image-based approaches after genetic perturbations is a fundamental tool to decipher genotype–phenotype relationships. This can be done in a high-throughput manner in an arrayed format, whereby cells are plated into individual wells and subjected to a particular genetic perturbation, drug treatment or other stimulus. High-throughput imaging can then be used to document cellular phenotypes in individual wells to understand how specific phenotypes arise from specific genetic perturbations. In kinetoplastids, arrayed image-based screening has only been used to study the phenotypic effects of perturbations on a relatively small numbers of genes [17, 35, 45, 115–119]. In mammalian cells and yeast, large scale and genome wide arrayed image-based screens have been used to phenotype genetic perturbations to identify regulators of endocytosis [120, 121], lipid droplet formation [122], ER targeting [123], nuclear condensate integrity [124], and profile the localizations of many thousands of pathogenic protein variants [125].

An arrayed image-based screen has recently been reported in *T. gondii* [41]. Here, Li *et al.* performed a CRISPR–Cas9 KO screen in cells with fluorescently labelled actin and an apicoplast marker to identify genes which may be involved in actin dynamics, apicoplast segregation, and egress. This study used a split Cas9 design, allowing inducible gene KO upon addition of rapamycin. In total, the authors selected 320 genes for deletion and were able to identify 42 genes which resulted in detectable phenotypic differences in the above criteria. Readout of arrayed screening is straightforward as the identity of each perturbation is linked to each well. Although these screens provide immensely powerful resources and can be used to discover complex cell biology, they are more applicable when used for targeted small-scale screens. Applying such arrayed image-based screens at the genome wide level for most laboratories is not technically feasible.

**Figure 4.**
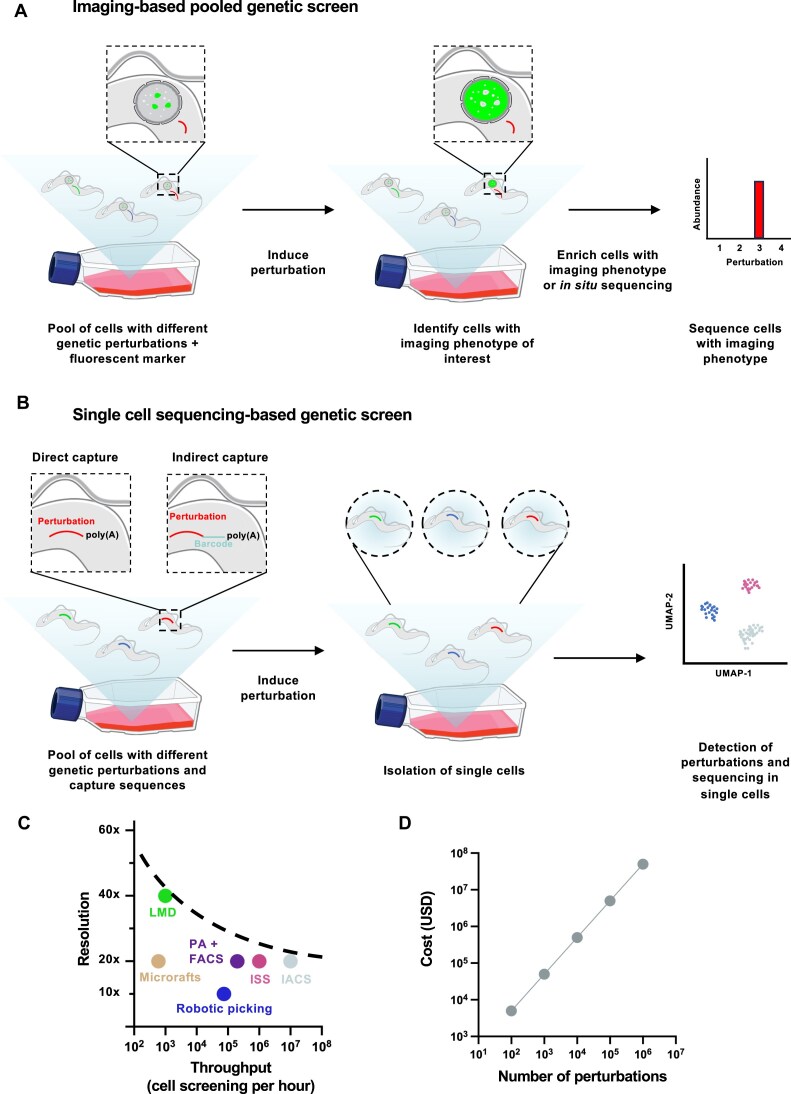
Image-based and single-cell sequencing-based pooled genetic screens. (**A**) Workflow for performing a pooled image-based genetic screen. A pool of cells is generated which each contain a different genetic perturbation. These cells also contain a fluorescent marker to visualize cellular DNA, RNA, protein, or other marker of interest. The perturbation library is then induced and cells with an imaging phenotype of interest are either isolated and enriched or perturbation signatures are sequenced *in situ*. (**B**) Workflow for performing pooled single-cell sequencing-based genetic screen. A pool of cells is generated in which the perturbations of interest are modified such that they can be directly or indirectly captured by poly(A) enrichment for subsequent single-cell sequencing. After inducing perturbations and isolating single cells, cells are sequenced to obtain single-cell information for each perturbation. (**C**) Comparison of the different technologies which can be used to isolate, enrich or directly sequence cells from pooled genetic screens based on imaging phenotypes. The maximum reported resolution and the approximate throughput (for mammalian cells) are shown for each method. The dotted line indicates the technical space which has currently been developed for pooled image-based screens. LMD, laser-assisted microdissection; PA + FACS, photoactivation + FACS; ISS, *in situ* sequencing; IACS, image-activated cell sorting. (**D**) Estimation of cost for scRNA-seq for increasing numbers of perturbations. Cost were calculated assuming 100 transcriptomes per perturbation, with a read depth of 50 000 reads per cell and an average cost per cell (reagents and sequencing) of 0.5$ using a 10x-Chromium platform.

However, in the last few years pooled image-based screening has emerged as a viable alternative to arrayed image-based screening. Instead of plating cells into individual wells, pools of cells are assessed for image-based phenotypes, and specific perturbations are determined by isolation and enrichment of cells of interest followed by sequencing, or, *in situ* genotyping [126] (Fig. 4A). Several methods for selective isolation of cells from pooled genetic screens based on imaging phenotypes have been developed in the last few years. These include selective photoactivation with fluorescence-activated cell sorting (FACS) [127, 128], laser-assisted microdissection [129], robotic cell picking [130], microrafts [131], and image-activated cell sorting [132, 133]. Several of these technologies have been combined with CRISPR screening to identify factors which mediate transcription factor nuclear translocation [128, 133], autophagy [129], nuclear size [127], stress granule formation [131], and organelle targeting [128] (Table 3). For a comprehensive review on phenotypic cell enrichment from pooled genetic screens, see [126, 134]. One of the key benefits of these pooled image-based screens over arrayed formats is that the throughput of analysis can be increased by several orders of magnitude (Fig. 3), thus readily facilitating genome wide phenotypic screens. In addition, methods to measure perturbation barcodes *in situ* have been developed and combined with CRISPR screening in human cells to carry out image-based phenotypic profiling of perturbations to thousands of genes at the throughput of tens of millions of single cells [135, 136].

**Table 3.** Overview of major technologies used to perform pooled image-based genetic screens performed in mammalian systems. The schematics used for the cells in this table were adapted from [189]

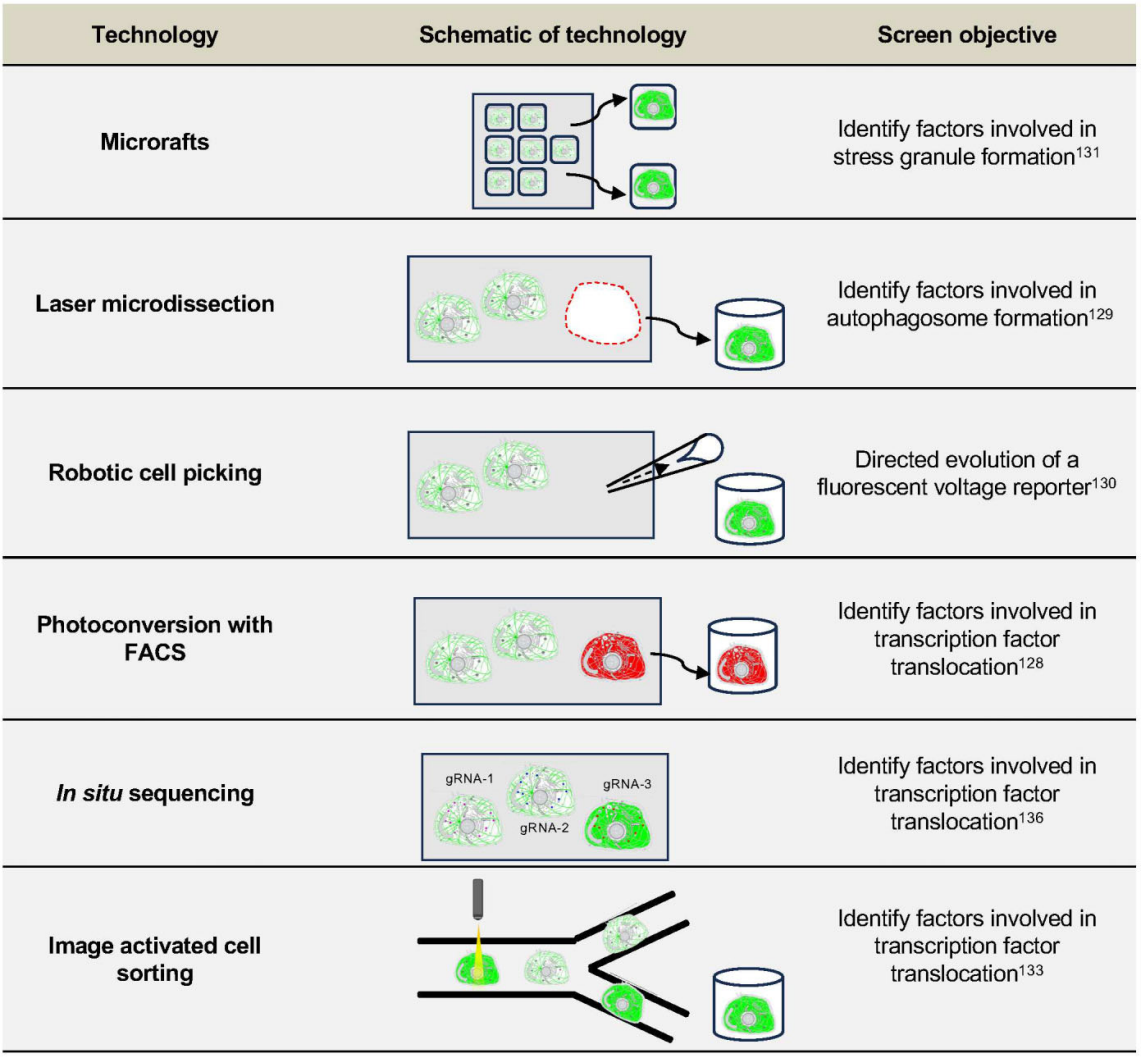

Currently, pooled image-based screens have primarily been used in mammalian systems. Many of these technologies are still limited by trade-offs with resolution over throughput, technical difficulties in implementation, cost, and lack of readily available equipment (Fig. 4C). In the future, as pooled genetic screens are developed, optimized and disseminated in parasitology research, the urge to implement pooled image-based screening will no doubt increase significantly.

## Pooled genetic screens with scRNA-seq readout

High-throughput RNA sequencing (RNA-seq) has been (and continues to be) instrumental in developing our understanding of parasite cell biology [70, 137–140]. However, RNA-seq is a bulk method to analyse transcriptomes, producing averaged transcriptional profiles across cell populations. Therefore, cell-to-cell heterogeneity cannot be discriminated using this technique. Cellular heterogeneity underpins several biological processes in protozoan parasites such as host adaptation, developmental progression and antigenic variation [141]. This has spurred the application and development of single-cell RNA sequencing (scRNA-seq) technologies which now allows us to uncover previously undetectable cell populations. A variety of both droplet-based (inDrop [142], Chromium [143]) and plate-based (Smart-seq2 [144, 145], SL-Smart-seq3xpress [146, 147]) scRNA-seq techniques have now been successfully applied to *T. brucei*. Combined, these scRNA-seq technologies have been used to study antigenic variation [142, 144, 148], developmental progression [145, 149], cell cycle regulation [143, 150], and quorum sensing [150]. For a further overview of scRNA-seq applications in kinetoplastids see [151].

Another powerful approach to study gene function is to combine scRNA-seq with genetic perturbations to determine the transcriptomes of single cells after knockdown or knockout of a specific genes. This has the ability to uncover phenotypes which are masked by bulk RNA-seq experiments. For example, a recent study performed scRNA-seq on cells in which the proteins VEX1 or VEX2 were knocked down by RNAi [148]. Previously, bulk RNA-seq experiments had been used to show that VEX1/VEX2 knockdown results in the expression of multiple VSG genes, indicating these proteins regulate monogenic VSG expression [152]. However, from these experiments it was unclear how many different VSGs can be expressed simultaneously in single cells upon knockdown of VEX1/VEX2. Using scRNA-seq, it was found that cells can express up to 12 VSGs simultaneously, with the majority (80%) expressing 3–6 following knockdown of VEX2 [148]. Another recent study performed scRNA-seq in insect-stage *T. brucei* cells upon knockdown of DRBD18 (an RNA-binding protein involved in repressing transcripts expressed in other life cycle stages). Here, the authors were able to show that DRBD18 selects poly(A) sites which result in splicing events that retain destabilization elements in developmentally regulated mRNAs to decrease their expression [153]. Importantly, the scRNA-seq analysis conducted here allowed the authors to precisely correlate 3′UTR length with changes in mRNA expression levels. Although a previous bulk RNA-seq experiment on DRBD18 RNAi cells had identified alterations to 3′UTR length, it did not capture the full scale of heterogeneity in poly(A) site selection or how this was correlated with expression level [154].

Therefore, single-cell transcriptomics of genetically perturbed cells has the potential to reveal phenotypes that are masked by bulk analyses. Historically, most scRNA-seq perturbation studies focused on individual genes [155–160]. However, recent advancements have introduced technologies enabling scRNA-seq-based profiling from pooled genetic screens. These innovations make it possible to assess thousands of perturbation transcriptomes simultaneously in a single experiment [161–164].

Commonly used approaches in single-cell perturbation screens include CROP-seq [165], CRISP-seq [166], Perturb-seq [167], direct capture Perturb-seq [168], and Mosaic-seq [169]. For a comprehensive overview on combining scRNA-seq technologies with CRISPR screens see [170]. A universal feature of these methods is the need for specific capture of the sgRNA used for genetic perturbation. This necessity arises because sgRNAs are typically transcribed by polymerases that do not produce polyadenylated RNA. Since scRNA-seq approaches rely on polyT primers to enrich for mRNA during cDNA synthesis, alternative strategies are required to capture sgRNAs effectively. These methods address this challenge by either (i) creating a secondary polyadenylated sgRNA transcript by embedding it in the 3′UTR of another gene, (ii) modifying the *trans*-activating crRNA (tracrRNA) to enable specific capture and amplification of sgRNAs, or (iii) incorporating a barcode in the UTR of a gene adjacent to the sgRNA, which is linked via NGS of the plasmid library prior to transfection (Fig. 4B).

Of these approaches, direct capture Perturb-seq has recently been used in *T. gondii* [171]. Here the authors performed a pooled CRISPR screen measuring host cell and parasite single-cell transcriptomes after knockout of 256 *T. gondii* proteins (5 sgRNAs each) which are secreted into the host cell. This approach appeared to be sensitive at detecting sgRNAs, with the sgRNA identified in ∼75% of cells. Further, the authors were able to identify several proteins which alter host cell transcription and further characterized two of these effectors in detail. Importantly, previous pooled CRISPR knockout screens analysing only cell fitness as the readout failed to identify many proteins known to modulate host cell transcription [172]. Again, this study indicates that scRNA-seq can be used to uncover phenotypes which are masked by bulk analyses in pooled genetic screens.

However, it should be noted that the cost of such screens is currently prohibitive when applied at the scale of thousands of genes (Fig. 4D). Expected future developments in scRNA-seq platforms that reduce cost and increase sensitivity and scalability will allow pooled genetic screens to be combined with single-cell transcriptomic readouts at the genome wide level (Fig. 3).

## Conclusions

Genetic screens have the ability to uncover unanticipated and novel cell biology, characterize entire biological pathways, assign gene function, determine mechanisms of drug resistance, and provide large scale resources for the research community. Key future challenges will be developing and optimizing genetic screens in kinetoplastids (particularly pooled genetic screens) using different perturbation strategies and combining the information readout from such screens by imaging, scRNA-seq and other “omics” technologies.

As highlighted in this review, CRISPR–Cas technologies are ideally suited for genetic screens in that they can be modified/engineered to be used for all types of perturbation, they are highly efficient, many are compatible with pooled genetic screening and they only require a sgRNA which can easily be cloned as plasmid libraries using oligo pools. In addition, CRISPR–Cas technologies have further application beyond genetic screens to address various biological questions. For instance, CRISPR–Cas9 has recently been used by our laboratory and others to determine mechanisms of antigenic variation by VSG switching in *T. brucei* [146, 173]. In these studies, Cas9 was used to explore the consequences of introducing specific DSBs in the active VSG gene. Following DSB induction, VSG switching was shown to occur via segmental gene conversion if a VSG containing a homologous region was present in the genome. If no VSG homolog was present, DNA resection was shown to occur to mediate break induced replication with a conserved sequence present in all VSG transcription units. These experiments shed light on antigenic variation and in particular antigen switching hierarchies, both long appreciated but incompletely understood processes observed in many organisms which utilize antigenic variation [144, 174, 175]. Importantly in these studies, CRISPR–Cas9 allowed specific DSB induction in a range of unmodified native positions in the active VSG transcription unit. Prior to this, selective DSB induction had only been possible with prior genetic modification to insert a recognition sequence for the homing endonuclease I-SceI, meaning specific cutting in the active VSG gene was not possible [176, 177].

Furthermore, new methods for performing genetic perturbations are becoming increasingly available through the discovery and engineering of novel CRISPR systems and RNA-guided genome editing systems [178, 179]. In addition to CRISPR–Cas base editors and nucleases, CRISPR–Cas integrases, transposases, prime editors, and novel programable RNA-guided transposases have emerged as new tools for genome engineering [180–184]. However, many of these technologies have only recently been discovered and have not been used in large scale genetic screens. Importantly, many of these systems do not require repair templates (or can be used with universal repair templates), meaning that they will be applicable to pooled genetic screens in parasites lacking NHEJ to increase scalability for large scale and whole genome screens. Adapting and optimizing these technologies for usage in kinetoplastid research will be a key future challenge.

Another key future challenge will be developing image-based pooled genetic screens in kinetoplastids. Image-based genetic screens have the power to identify genes involved in complex cellular phenotypes which cannot be found through conventional cell fitness-based genetic screens. For example, image-based screens can be used to identify genes involved in regulating protein translocation, organelle structure, protein co-localization, and biological processes which do not significantly affect cell fitness when perturbed. Determining optimal imaging and cell enrichment approaches which can sufficiently factor throughput, resolution, sensitivity, and cost will be critical to successfully apply image-based genetic screens in kinetoplastids.

Although many types of large-scale genetic screen have been performed in *T. brucei* and *Leishmania*, no whole genome or large-scale genetic screens have been performed in *T. cruzi* (Fig. 3). This is primarily due to relatively low transfection efficiency, lack of RNAi, slower growth, and lower quality of genome assemblies. Technological advances that increase transfection efficiency and genetic tools that can be used in pooled formats are needed before large-scale genetic screens can be performed in this organism.

Lastly, combining scRNA-seq, other single-cell “omics” technologies and imaging approaches with genetic screens will be a future challenge [185]. Although scRNA-seq based readout of CRISPR screening has been demonstrated in *T. gondii*, it remains to be seen whether this or other single-cell sequencing technologies can be combined with genetic screens in kinetoplastid organisms. Furthermore, adapting multimodal single-cell sequencing technologies that can simultaneously measure multiple parameters in perturbed cells, such as profiling chromatin accessibility, protein levels or other gene expression related phenotypes, will facilitate understanding of how many (or all) genes in an organism affect these different layers of biological function [186]. Selecting appropriate single-cell sequencing approaches which give sufficient sensitivity, specificity, scalability, and cost effectiveness will be necessary for combining single-cell sequencing with genetic screens.

With the increasing number of available whole genome perturbation screens, subcellular localization atlases and scRNA-seq datasets, resources that integrate these for exploration, such as those provided by VEuPathDB [187, 188], are critical to the research community. Indeed, as has been demonstrated in multiple studies discussed in this review, mining these available datasets can be extremely useful for narrowing down candidates for targeted genetic screens without the need to perform a whole genome screen (Table 2). The importance of these resources will only increase further as imaging and single-cell sequencing readouts add a new dimension to high-throughput genetic screens in kinetoplastids and beyond.

## Data Availability

No new data were generated in support of this review.

## References

[1] Cong L, Ran FA, Cox D et al. Multiplex genome engineering using CRISPR–Cas systems. Science. 2013; 339:819–23. 10.1126/science.1231143. 23287718 PMC3795411

[2] Clayton C Regulation of gene expression in trypanosomatids: living with polycistronic transcription. Open Biol. 2019; 9:190072 10.1098/rsob.190072. 31164043 PMC6597758

[3] Bock C, Datlinger P, Chardon F et al. High-content CRISPR screening. Nat Rev Methods Primers. 2022; 2:8 10.1038/s43586-021-00093-4. PMC10200264 37214176

[4] Borst P, Rudenko G Antigenic variation in African trypanosomes. Science. 1994; 264:1872–3. 10.1126/science.7516579. 7516579

[5] Rojas F, Silvester E, Young J et al. Oligopeptide signaling through TbGPR89 drives trypanosome quorum sensing. Cell. 2019; 176:306–17. 10.1016/j.cell.2018.10.041. 30503212 PMC6333907

[6] Bastin P, Sherwin T, Gull K Paraflagellar rod is vital for trypanosome motility. Nature. 1998; 391:548 10.1038/35300. 9468133

[7] Kooter JM, Borst P Alpha-amanitin-insensitive transcription of variant surface glycoprotein genes provides further evidence for discontinuous transcription in trypanosomes. Nucleic Acids Res. 1984; 12:9457–72. 10.1093/nar/12.24.9457. 6514580 PMC320473

[8] Agabian N Trans splicing of nuclear pre-mRNAs. Cell. 1990; 61:1157–60. 10.1016/0092-8674(90)90674-4. 2142018

[9] Kachale A, Pavlikova Z, Nenarokova A et al. Short tRNA anticodon stem and mutant eRF1 allow stop codon reassignment. Nature. 2023; 613:751–8. 10.1038/s41586-022-05584-2. 36631608

[10] Yin JA, Frick L, Scheidmann MC et al. Arrayed CRISPR libraries for the genome-wide activation, deletion and silencing of human protein-coding genes. Nat Biomed Eng. 2025; 9:127–48. 10.1038/s41551-024-01278-4. 39633028 PMC11754104

[11] Faria J, Luzak V, Muller LSM et al. Spatial integration of transcription and splicing in a dedicated compartment sustains monogenic antigen expression in African trypanosomes. Nat Microbiol. 2021; 6:289–300. 10.1038/s41564-020-00833-4. 33432154 PMC7610597

[12] Budzak J, Jones R, Tschudi C et al. An assembly of nuclear bodies associates with the active VSG expression site in African trypanosomes. Nat Commun. 2022; 13:101 10.1038/s41467-021-27625-6. 35013170 PMC8748868

[13] Ngo H, Tschudi C, Gull K et al. Double-stranded RNA induces mRNA degradation in Trypanosoma brucei. Proc Natl Acad Sci USA. 1998; 95:14687–92. 10.1073/pnas.95.25.14687. 9843950 PMC24510

[14] Wirtz E, Clayton C Inducible gene expression in trypanosomes mediated by a prokaryotic repressor. Science. 1995; 268:1179–83. 10.1126/science.7761835. 7761835

[15] Morris JC, Wang Z, Drew ME et al. Glycolysis modulates trypanosome glycoprotein expression as revealed by an RNAi library. EMBO J. 2002; 21:4429–38. 10.1093/emboj/cdf474. 12198145 PMC125414

[16] Boutros M, Kiger AA, Armknecht S et al. Genome-wide RNAi analysis of growth and viability in Drosophila cells. Science. 2004; 303:832–5. 10.1126/science.1091266. 14764878

[17] Subramaniam C, Veazey P, Redmond S et al. Chromosome-wide analysis of gene function by RNA interference in the African trypanosome. Euk Cell. 2006; 5:1539–49. 10.1128/EC.00141-06. PMC1563588 16963636

[18] Glover L, Horn D Site-specific DNA double-strand breaks greatly increase stable transformation efficiency in *Trypanosoma brucei*. Mol Biochem Parasitol. 2009; 166:194–7. 10.1016/j.molbiopara.2009.03.010. 19459229 PMC2691778

[19] Alsford S, Turner DJ, Obado SO et al. High-throughput phenotyping using parallel sequencing of RNA interference targets in the African trypanosome. Genome Res. 2011; 21:915–24. 10.1101/gr.115089.110. 21363968 PMC3106324

[20] Bachmaier S, Gould MK, Polatoglou E et al. Novel kinetoplastid-specific cAMP binding proteins identified by RNAi screening for cAMP resistance in *Trypanosoma brucei*. Front Cell Infect Microbiol. 2023; 13:1204707 10.3389/fcimb.2023.1204707. 37475965 PMC10354285

[21] Alsford S, Eckert S, Baker N et al. High-throughput decoding of antitrypanosomal drug efficacy and resistance. Nature. 2012; 482:232–6. 10.1038/nature10771. 22278056 PMC3303116

[22] Davies C, Ooi CP, Sioutas G et al. TbSAP is a novel chromatin protein repressing metacyclic variant surface glycoprotein expression sites in bloodstream form *Trypanosoma brucei*. Nucleic Acids Res. 2021; 49:3242–62. 10.1093/nar/gkab109. 33660774 PMC8034637

[23] Marques CA, Ridgway M, Tinti M et al. Genome-scale RNA interference profiling of *Trypanosoma brucei* cell cycle progression defects. Nat Commun. 2022; 13:5326 10.1038/s41467-022-33109-y. 36088375 PMC9464253

[24] Mony BM, MacGregor P, Ivens A et al. Genome-wide dissection of the quorum sensing signalling pathway in *Trypanosoma brucei*. Nature. 2014; 505:681–5. 10.1038/nature12864. 24336212 PMC3908871

[25] Horn D Genome-scale RNAi screens in African trypanosomes. Trends Parasitol. 2022; 38:160–73. 10.1016/j.pt.2021.09.002. 34580035

[26] Ullu E, Tschudi C, Chakraborty T RNA interference in protozoan parasites. Cell Microbiol. 2004; 6:509–19. 10.1111/j.1462-5822.2004.00399.x. 15104593

[27] Abudayyeh OO, Gootenberg JS, Essletzbichler P et al. RNA targeting with CRISPR–Cas13. Nature. 2017; 550:280–4. 10.1038/nature24049. 28976959 PMC5706658

[28] Wessels HH, Mendez-Mancilla A, Hao Y et al. Efficient combinatorial targeting of RNA transcripts in single cells with Cas13 RNA perturb-seq. Nat Methods. 2023; 20:86–94. 10.1038/s41592-022-01705-x. 36550277 PMC10030154

[29] Cruz A, Beverley SM Gene replacement in parasitic protozoa. Nature. 1990; 348:171–3. 10.1038/348171a0. 2234081

[30] Santos R, Silva GLA, Santos EV et al. A DiCre recombinase-based system for inducible expression in Leishmania major. Mol Biochem Parasitol. 2017; 216:45–8. 10.1016/j.molbiopara.2017.06.006. 28629935

[31] Kim HS, Li Z, Boothroyd C et al. Strategies to construct null and conditional null trypanosoma brucei mutants using cre-recombinase and loxP. Mol Biochem Parasitol. 2013; 191:16–9. 10.1016/j.molbiopara.2013.08.001. 23954366 PMC3830529

[32] Asencio C, Herve P, Morand P et al. Streptococcus pyogenes Cas9 ribonucleoprotein delivery for efficient, rapid and marker-free gene editing in Trypanosoma and Leishmania. Mol Microbiol. 2024; 121:1079–94. 10.1111/mmi.15256. 38558208

[33] Rico E, Jeacock L, Kovarova J et al. Inducible high-efficiency CRISPR–Cas9-targeted gene editing and precision base editing in African trypanosomes. 2018; 8:7960 10.1038/s41598-018-26303-w. PMC5962531 29785042

[34] Vasquez JJ, Wedel C, Cosentino RO et al. Exploiting CRISPR–Cas9 technology to investigate individual histone modifications. Nucleic Acids Res. 2018; 46:e106 10.1093/nar/gky517. 29912461 PMC6182134

[35] Lander N, Li ZH, Niyogi S et al. CRISPR–Cas9-induced disruption of paraflagellar rod protein 1 and 2 genes in Trypanosoma cruzi reveals their role in flagellar attachment. mBio. 2015; 6:e01012 10.1128/mBio.01012-15. 26199333 PMC4513075

[36] Peng D, Kurup SP, Yao PY et al. CRISPR–Cas9-mediated single-gene and gene family disruption in *Trypanosoma cruzi*. mBio. 2014; 6:e02097-14 10.1128/mBio.02097-14. 25550322 PMC4281920

[37] Schadeli D, Serricchio M, Hamidane HB et al. Cardiolipin depletion-induced changes in the *Trypanosoma brucei* proteome. FASEB J. 2019; 33:13161–75. 10.1096/fj.201901184RR. 31536395

[38] Beneke T, Madden R, Makin L et al. A CRISPR Cas9 high-throughput genome editing toolkit for kinetoplastids. R Soc Open Sci. 2017; 4:170095 10.1098/rsos.170095. 28573017 PMC5451818

[39] Beneke T, Gluenz E LeishGEdit: a method for rapid gene knockout and tagging using CRISPR–Cas9. Methods Mol Biol. 2019; 1971:189–210. 30980304 10.1007/978-1-4939-9210-2_9

[40] Duncan SM, Myburgh E, Philipon C et al. Conditional gene deletion with DiCre demonstrates an essential role for CRK3 in Leishmania mexicana cell cycle regulation. Mol Microbiol. 2016; 100:931–44. 10.1111/mmi.13375. 26991545 PMC4913733

[41] Li W, Grech J, Stortz JF et al. A splitCas9 phenotypic screen in *Toxoplasma gondii* identifies proteins involved in host cell egress and invasion. Nat Microbiol. 2022; 7:882–95. 10.1038/s41564-022-01114-y. 35538310

[42] Wang T, Birsoy K, Hughes NW et al. Identification and characterization of essential genes in the human genome. Science. 2015; 350:1096–101. 10.1126/science.aac7041. 26472758 PMC4662922

[43] Sidik SM, Huet D, Ganesan SM et al. A genome-wide CRISPR screen in toxoplasma identifies essential apicomplexan genes. Cell. 2016; 166:1423–35. 10.1016/j.cell.2016.08.019. 27594426 PMC5017925

[44] Leish GEMT LeishGEM: genome-wide deletion mutant fitness and protein localisations in Leishmania. Trends Parasitol. 2024; 40:675–8. 39030136 10.1016/j.pt.2024.06.003

[45] Beneke T, Demay F, Hookway E et al. Genetic dissection of a Leishmania flagellar proteome demonstrates requirement for directional motility in sand fly infections. PLoS Pathog. 2019; 15:e1007828 10.1371/journal.ppat.1007828. 31242261 PMC6615630

[46] Baker N, Catta-Preta CMC, Neish R et al. Systematic functional analysis of leishmania protein kinases identifies regulators of differentiation or survival. Nat Commun. 2021; 12:1244 10.1038/s41467-021-21360-8. 33623024 PMC7902614

[47] Roberts AJ, Ong HB, Clare S et al. Systematic identification of genes encoding cell surface and secreted proteins that are essential for *in vitro* growth and infection in Leishmania donovani. PLoS Pathog. 2022; 18:e1010364 10.1371/journal.ppat.1010364. 35202447 PMC8903277

[48] Albuquerque-Wendt A, McCoy C, Neish R et al. TransLeish: Identification of membrane transporters essential for survival of intracellular Leishmaniaparasites in a systematic gene deletion screen. Nat Commun. 2025; 16:299 10.1038/s41467-024-55538-7. 39747086 PMC11696137

[49] Queffeulou M, Leprohon P, Fernandez-Prada C et al. CRISPR–Cas9 high-throughput screening to study drug resistance in Leishmania infantum. mBio. 2024; 15:e0047724 10.1128/mbio.00477-24. 38864609 PMC11253630

[50] Zhang WW, Matlashewski G Single-strand annealing plays a major role in double-strand DNA break repair following CRISPR–Cas9 cleavage in Leishmania. mSphere. 2019; 4:e00408-19 10.1128/mSphere.00408-19. 31434745 PMC6706467

[51] Lue NZ, Liau BB Base editor screens for *in situ*mutational scanning at scale. Mol Cell. 2023; 83:2167–87. 10.1016/j.molcel.2023.06.009. 37390819 PMC10330937

[52] Kuscu C, Parlak M, Tufan T et al. CRISPR–STOP: gene silencing through base-editing-induced nonsense mutations. Nat Methods. 2017; 14:710–2. 10.1038/nmeth.4327. 28581493

[53] Hanna RE, Hegde M, Fagre CR et al. Massively parallel assessment of human variants with base editor screens. Cell. 2021; 184:1064–80. 10.1016/j.cell.2021.01.012. 33606977

[54] Engstler M, Beneke T Gene editing and scalable functional genomic screening in Leishmania species using the CRISPR–Cas9 cytosine base editor toolbox LeishBASEedit. eLife. 2023; 12:e85605 10.7554/eLife.85605. 37222701 PMC10208639

[55] Zhang X, Chen L, Zhu B et al. Increasing the efficiency and targeting range of cytidine base editors through fusion of a single-stranded DNA-binding protein domain. Nat Cell Biol. 2020; 22:740–50. 10.1038/s41556-020-0518-8. 32393889

[56] Kim YB, Komor AC, Levy JM et al. Increasing the genome-targeting scope and precision of base editing with engineered Cas9-cytidine deaminase fusions. Nat Biotechnol. 2017; 35:371–6. 10.1038/nbt.3803. 28191901 PMC5388574

[57] Lykke-Andersen S, Jensen TH Nonsense-mediated mRNA decay: an intricate machinery that shapes transcriptomes. Nat Rev Mol Cell Biol. 2015; 16:665–77. 10.1038/nrm4063. 26397022

[58] Cosentino RO, Brink BG, Siegel TN Allele-specific assembly of a eukaryotic genome corrects apparent frameshifts and reveals a lack of nonsense-mediated mRNA decay. NAR Genom Bioinform. 2021; 3:lqab082 10.1093/nargab/lqab082. 34541528 PMC8445201

[59] Herrmann May N, Cao A, Schmid A et al. Improved base editing and functional screening in Leishmania via co-expression of the AsCas12a ultra variant, a T7 RNA polymerase, and a cytosine base editor. eLife. 2025; 13:RP97437 10.7554/eLife.97437. 39991929 PMC11850003

[60] Sopko R, Huang D, Preston N et al. Mapping pathways and phenotypes by systematic gene overexpression. Mol Cell. 2006; 21:319–30. 10.1016/j.molcel.2005.12.011. 16455487

[61] Shahi SK, Krauth-Siegel RL, Clayton CE Overexpression of the putative thiol conjugate transporter TbMRPA causes melarsoprol resistance in *Trypanosoma brucei*. Mol Microbiol. 2002; 43:1129–38. 10.1046/j.1365-2958.2002.02831.x. 11918801

[62] Rajao MA, Furtado C, Alves CL et al. Unveiling benznidazole’s mechanism of action through overexpression of DNA repair proteins in *Trypanosoma cruzi*. Environ and Mol Mutagen. 2014; 55:309–21. 10.1002/em.21839. 24347026

[63] Begolo D, Erben E, Clayton C Drug target identification using a trypanosome overexpression library. Antimicrob Agents Chemother. 2014; 58:6260–4. 10.1128/AAC.03338-14. 25049244 PMC4187942

[64] Gazanion E, Fernandez-Prada C, Papadopoulou B et al. Cos-Seq for high-throughput identification of drug target and resistance mechanisms in the protozoan parasite Leishmania. Proc Natl Acad Sci USA. 2016; 113:E3012–21. 10.1073/pnas.1520693113. 27162331 PMC4889358

[65] Carter M, Gomez S, Gritz S et al. A trypanosoma brucei ORFeome-based gain-of-function library identifies genes that promote survival during melarsoprol treatment. mSphere. 2020; 5:e00769-20 10.1128/mSphere.00769-20. 33028684 PMC7568655

[66] Wall RJ, Rico E, Lukac I et al. Clinical and veterinary trypanocidal benzoxaboroles target CPSF3. Proc Natl Acad Sci USA. 2018; 115:9616–21. 10.1073/pnas.1807915115. 30185555 PMC6156652

[67] Erben ED, Fadda A, Lueong S et al. A genome-wide tethering screen reveals novel potential post-transcriptional regulators in *Trypanosoma brucei*. PLoS Pathog. 2014; 10:e1004178 10.1371/journal.ppat.1004178. 24945722 PMC4055773

[68] Kampmann M CRISPRi and CRISPRa screens in mammalian cells for precision biology and medicine. ACS Chem Biol. 2018; 13:406–16. 10.1021/acschembio.7b00657. 29035510 PMC5886776

[69] Schmidt R, Steinhart Z, Layeghi M et al. CRISPR activation and interference screens decode stimulation responses in primary human T cells. Science. 2022; 375:eabj4008 10.1126/science.abj4008. 35113687 PMC9307090

[70] Siegel TN, Hekstra DR, Wang X et al. Genome-wide analysis of mRNA abundance in two life-cycle stages of *Trypanosoma brucei* and identification of splicing and polyadenylation sites. Nucleic Acids Res. 2010; 38:4946–57. 10.1093/nar/gkq237. 20385579 PMC2926603

[71] Luzak V, Osses E, Danese A et al. SLAM-seq reveals independent contributions of RNA processing and stability to gene expression in African trypanosomes. Nucleic Acids Res. 2025; 53:gkae1203 10.1093/nar/gkae1203. 39673807 PMC11797058

[72] Cordon-Obras C, Gomez-Linan C, Torres-Rusillo S et al. Identification of sequence-specific promoters driving polycistronic transcription initiation by RNA polymerase II in trypanosomes. Cell Rep. 2022; 38:110221 10.1016/j.celrep.2021.110221. 35021094

[73] Wedel C, Forstner KU, Derr R et al. GT-rich promoters can drive RNA pol II transcription and deposition of H2A.Z in African trypanosomes. 2017; 36:2581–94. 10.15252/embj.201695323. PMC5579346 28701485

[74] Cao C, Li A, Xu C et al. Enhancement of protein translation by CRISPR–dCasRx coupled with SINEB2 repeat of noncoding RNAs. Nucleic Acids Res. 2023; 51:e33 10.1093/nar/gkad010. 36715335 PMC10085674

[75] Otoupal PB, Cress BF, Doudna JA et al. CRISPR–RNAa: targeted activation of translation using dCas13 fusions to translation initiation factors. Nucleic Acids Res. 2022; 50:8986–98. 10.1093/nar/gkac680. 35950485 PMC9410913

[76] Padilla A, Noiva R, Lee N et al. An atypical protein disulfide isomerase from the protozoan parasite Leishmania containing a single thioredoxin-like domain. J Biol Chem. 2003; 278:1872–8. 10.1074/jbc.M210322200. 12427741

[77] Maric D, McGwire BS, Buchanan KT et al. Molecular determinants of ciliary membrane localization of *Trypanosoma cruzi*flagellar calcium-binding protein. J Biol Chem. 2011; 286:33109–17. 10.1074/jbc.M111.240895. 21784841 PMC3190917

[78] Leal S, Acosta-Serrano A, Morris J et al. Transposon mutagenesis of *Trypanosoma brucei* identifies glycosylation mutants resistant to concanavalin A. J Biol Chem. 2004; 279:28979–88. 10.1074/jbc.M403479200. 15123607

[79] Bhattacharya A, Leprohon P, Bigot S et al. Coupling chemical mutagenesis to next generation sequencing for the identification of drug resistance mutations in Leishmania. Nat Commun. 2019; 10:5627 10.1038/s41467-019-13344-6. 31819054 PMC6901541

[80] Kolev NG, Ramey-Butler K, Cross GA et al. Developmental progression to infectivity in *Trypanosoma brucei* triggered by an RNA-binding protein. Science. 2012; 338:1352–3. 10.1126/science.1229641. 23224556 PMC3664091

[81] Rojas-Sanchez S, Kolev NG, Tschudi C Deep mutational scanning of the *Trypanosoma brucei* developmental regulator RBP6 reveals an essential disordered region influenced by positive residues. Nat Commun. 2025; 16:1168 10.1038/s41467-025-56553-y. 39885181 PMC11782513

[82] McDermott SM, Pham V, Oliver B et al. Deep mutational scanning of the RNase III-like domain in *Trypanosoma brucei* RNA editing protein KREPB4. Front Cell Infect Microbiol. 2024; 14:1381155 10.3389/fcimb.2024.1381155. 38650737 PMC11033214

[83] Trenaman A, Tinti M, Wall RJ et al. Post-transcriptional reprogramming by thousands of mRNA untranslated regions in trypanosomes. Nat Commun. 2024; 15:8113 10.1038/s41467-024-52432-0. 39285175 PMC11405848

[84] Kovarova J, Novotna M, Faria J et al. CRISPR–Cas9-based precision tagging of essential genes in bloodstream form African trypanosomes. Mol Biochem Parasitol. 2022; 249:111476 10.1016/j.molbiopara.2022.111476. 35378143

[85] Altmann S, Rico E, Carvalho S et al. Oligo targeting for profiling drug resistance mutations in the parasitic trypanosomatids. Nucleic Acids Res. 2022; 50:e79 10.1093/nar/gkac319. 35524555 PMC9371896

[86] Novotná M, Tinti M, Faria JRC et al. Precision-edited histone tails disrupt polycistronic gene expression controls in trypanosomes. bioRxiv21 March 2025, preprint: not peer reviewed 10.1101/2025.03.21.644654. PMC12227686 40615444

[87] Coelho MA, Cooper S, Strauss ME et al. Base editing screens map mutations affecting interferon-gamma signaling in cancer. Cancer Cell. 2023; 41:288–303. 10.1016/j.ccell.2022.12.009. 36669486 PMC9942875

[88] Akiyoshi B, Gull K Discovery of unconventional kinetochores in kinetoplastids. Cell. 2014; 156:1247–58. 10.1016/j.cell.2014.01.049. 24582333 PMC3978658

[89] Kelly S, Reed J, Kramer S et al. Functional genomics in *Trypanosoma brucei*: a collection of vectors for the expression of tagged proteins from endogenous and ectopic gene loci. Mol Biochem Parasitol. 2007; 154:103–9. 10.1016/j.molbiopara.2007.03.012. 17512617 PMC2705915

[90] Odenwald J, Gabiatti B, Braune S et al. Detection of TurboID fusion proteins by fluorescent streptavidin outcompetes antibody signals and visualises targets not accessible to antibodies. eLife. 2024; 13:RP95028 10.7554/eLife.95028.3. 39206942 PMC11361705

[91] Staneva DP, Carloni R, Auchynnikava T et al. A systematic analysis of *Trypanosoma brucei* chromatin factors identifies novel protein interaction networks associated with sites of transcription initiation and termination. Genome Res. 2021; 31:2138–54. 10.1101/gr.275368.121. 34407985 PMC8559703

[92] Dean S, Sunter J, Wheeler RJ et al. A toolkit enabling efficient, scalable and reproducible gene tagging in trypanosomatids. Open Biol. 2015; 5:140197 10.1098/rsob.140197. 25567099 PMC4313374

[93] Paterou A, Conde J, Týč J et al. A comprehensive toolkit for protein localization and functional analysis in trypanosomatids. Open Biol. 2025; 15:240361 10.1098/rsob.240361. 40169015 PMC11961264

[94] Carbajo CG, Han X, Savur B et al. A high-throughput protein tagging toolkit that retains endogenous untranslated regions for studying gene regulation in kinetoplastids. Open Biol. 2025; 15:240334 10.1098/rsob.240334. 39999874 PMC11858757

[95] Billington K, Halliday C, Madden R et al. Genome-wide subcellular protein map for the flagellate parasite *Trypanosoma b*rucei. Nat Microbiol. 2023; 8:533–47. 10.1038/s41564-022-01295-6. 36804636 PMC9981465

[96] Huh WK, Falvo JV, Gerke LC et al. Global analysis of protein localization in budding yeast. Nature. 2003; 425:686–91. 10.1038/nature02026. 14562095

[97] Cho NH, Cheveralls KC, Brunner AD et al. OpenCell: endogenous tagging for the cartography of human cellular organization. Science. 2022; 375:eabi6983 10.1126/science.abi6983. 35271311 PMC9119736

[98] Reicher A, Reinis J, Ciobanu M et al. Pooled multicolour tagging for visualizing subcellular protein dynamics. Nat Cell Biol. 2024; 26:745–56. 10.1038/s41556-024-01407-w. 38641660 PMC11098740

[99] Pyrih J, Hammond M, Alves A et al. Comprehensive sub-mitochondrial protein map of the parasitic protist *Trypanosoma brucei* defines critical features of organellar biology. Cell Rep. 2023; 42:113083 10.1016/j.celrep.2023.113083. 37669165

[100] Jeilani M, Billington K, Sunter JD et al. Nucleolar targeting in an early-branching eukaryote suggests a general mechanism for ribosome protein sorting. J Cell Sci. 2022; 135:jcs259701 10.1242/jcs.259701. 36052646 PMC9659390

[101] Ahmed M, Wheeler R, Tyc J et al. Identification of 30 transition fibre proteins in *Trypanosoma brucei* reveals a complex and dynamic structure. J Cell Sci. 2024; 137:jcs261692 10.1242/jcs.261692. 38572631 PMC11190437

[102] Sunter JD, Dean S, Wheeler RJ TrypTag.Org: from images to discoveries using genome-wide protein localisation in *Trypanosoma brucei*. Trends Parasitol. 2023; 39:328–31. 10.1016/j.pt.2023.02.008. 36925446

[103] Lopez-Escobar L, Hanisch B, Halliday C et al. Stage-specific transcription activator ESB1 regulates monoallelic antigen expression in *Trypanosoma brucei*. Nat Microbiol. 2022; 7:1280–90. 10.1038/s41564-022-01175-z. 35879525 PMC9352583

[104] Navarro M, Gull K A pol I transcriptional body associated with VSG mono-allelic expression in *Trypanosoma brucei*. Nature. 2001; 414:759–63. 10.1038/414759a. 11742402

[105] Budzak J, Rudenko G Pedal to the metal: nuclear splicing bodies turbo-charge VSG mRNA production in African trypanosomes. Front Cell Dev Biol. 2022; 10:876701 10.3389/fcell.2022.876701. 35517511 PMC9065277

[106] Halliday C, Dean S, Sunter JD et al. Subcellular protein localisation of *Trypanosoma brucei* bloodstream form-upregulated proteins maps stage-specific adaptations. Wellcome Open Res. 2023; 8:46 10.12688/wellcomeopenres.18586.2. 37251657 PMC10209625

[107] Serebrenik YV, Mani D, Maujean T et al. Pooled endogenous protein tagging and recruitment for systematic profiling of protein function. Cell Genomics. 2024; 4:100651 10.1016/j.xgen.2024.100651. 39255790 PMC11602618

[108] Nemcko F, Himmelsbach M, Loubiere V et al. Proteome-scale tagging and functional screening in mammalian cells by ORFtag. Nat Methods. 2024; 21:1668–1673. 10.1038/s41592-024-02339-x. 38969721 PMC11399080

[109] Valenti R, David Y, Edilbi D et al. A proteome-wide yeast degron collection for the dynamic study of protein function. J Cell Biol. 2025; 224:e202409050 10.1083/jcb.202409050. 39692734 PMC11654244

[110] Smith TA, Lopez-Perez GS, Herneisen AL et al. Screening the toxoplasma kinome with high-throughput tagging identifies a regulator of invasion and egress. Nat Microbiol. 2022; 7:868–81. 10.1038/s41564-022-01104-0. 35484233 PMC9167752

[111] Kimmel J, Schmitt M, Sinner A et al. Gene-by-gene screen of the unknown proteins encoded on *Plasmodium falciparum* chromosome 3. Cell Syst. 2023; 14:9–23 e27. 36657393 10.1016/j.cels.2022.12.001

[112] Ma Y, Weiss LM, Huang H Inducible suicide vector systems for *Trypanosoma cruzi*. Microbes Infect. 2015; 17:440–50. 10.1016/j.micinf.2015.04.003. 25899945

[113] Madeira da Silva L, Owens KL, Murta SM et al. Regulated expression of the Leishmania major surface virulence factor lipophosphoglycan using conditionally destabilized fusion proteins. Proc Natl Acad Sci USA. 2009; 106:7583–8. 10.1073/pnas.0901698106. 19383793 PMC2678649

[114] Gabiatti BP, Krenzer J, Braune S et al. Detailed characterisation of the trypanosome nuclear pore architecture reveals conserved asymmetrical functional hubs that drive mRNA export. PLoS Biol. 2025; 23:e3003024 10.1371/journal.pbio.3003024. 39899609 PMC11825100

[115] Duncan SM, Carbajo CG, Nagar R et al. Generation of a bloodstream form *Trypanosoma brucei* double glycosyltransferase null mutant competent in receptor-mediated endocytosis of transferrin. PLoS Pathog. 2024; 20:e1012333 10.1371/journal.ppat.1012333. 38935804 PMC11236118

[116] Vachova H, Alquicer G, Sedinova M et al. A rapid approach for in locus overexpression of *Trypanosoma brucei* proteins. Mol Biochem Parasitol. 2020; 239:111300 10.1016/j.molbiopara.2020.111300. 32682799

[117] Tavernelli LE, Motta MCM, Goncalves CS et al. Overexpression of *Trypanosoma cruz*i High Mobility Group B protein (TcHMGB) alters the nuclear structure, impairs cytokinesis and reduces the parasite infectivity. Sci Rep. 2019; 9:192 10.1038/s41598-018-36718-0. 30655631 PMC6336821

[118] Morales MA, Renaud O, Faigle W et al. Over-expression of Leishmania major MAP kinases reveals stage-specific induction of phosphotransferase activity. Int J Parasitol. 2007; 37:1187–99. 10.1016/j.ijpara.2007.03.006. 17481635

[119] Lander N, Chiurillo MA, Storey M et al. CRISPR–Cas9-mediated endogenous C-terminal tagging of *Trypanosoma cruzi* genes reveals the acidocalcisome localization of the inositol 1,4,5-trisphosphate receptor. J Biol Chem. 2016; 291:25505–15. 10.1074/jbc.M116.749655. 27793988 PMC5207250

[120] Collinet C, Stoter M, Bradshaw CR et al. Systems survey of endocytosis by multiparametric image analysis. Nature. 2010; 464:243–9. 10.1038/nature08779. 20190736

[121] Arlt H, Perz A, Ungermann C An overexpression screen in *Saccharomyces cerevisiae* identifies novel genes that affect endocytic protein trafficking. Traffic. 2011; 12:1592–603. 10.1111/j.1600-0854.2011.01252.x. 21777356

[122] Guo Y, Walther TC, Rao M et al. Functional genomic screen reveals genes involved in lipid-droplet formation and utilization. Nature. 2008; 453:657–61. 10.1038/nature06928. 18408709 PMC2734507

[123] Aviram N, Ast T, Costa EA et al. The SND proteins constitute an alternative targeting route to the endoplasmic reticulum. Nature. 2016; 540:134–8. 10.1038/nature20169. 27905431 PMC5513701

[124] Berchtold D, Battich N, Pelkmans L A systems-level study reveals regulators of membrane-less organelles in human cells. Mol Cell. 2018; 72:1035–49. 10.1016/j.molcel.2018.10.036. 30503769

[125] Lacoste J, Haghighi M, Haider S et al. Pervasive mislocalization of pathogenic coding variants underlying human disorders. Cell. 2024; 187:6725–41. 10.1016/j.cell.2024.09.003. 39353438 PMC11568917

[126] Walton RT, Singh A, Blainey PC Pooled genetic screens with image-based profiling. Mol Syst Biol. 2022; 18:e10768 10.15252/msb.202110768. 36366905 PMC9650298

[127] Yan X, Stuurman N, Ribeiro SA et al. High-content imaging-based pooled CRISPR screens in mammalian cells. J Cell Biol. 2021; 220:e202008158 10.1083/jcb.202008158. 33465779 PMC7821101

[128] Kanfer G, Sarraf SA, Maman Y et al. Image-based pooled whole-genome CRISPRi screening for subcellular phenotypes. J Cell Biol. 2021; 220:e202006180 10.1083/jcb.202006180. 33464298 PMC7816647

[129] Schmacke NA, Mädler SC, Wallmann G et al. SPARCS, a platform for genome-scale CRISPR screening for spatial cellular phenotypes. bioRxiv1 June 2023, preprint: not peer reviewed 10.1101/2023.06.01.542416.

[130] Piatkevich KD, Jung EE, Straub C et al. A robotic multidimensional directed evolution approach applied to fluorescent voltage reporters. Nat Chem Biol. 2018; 14:352–60. 10.1038/s41589-018-0004-9. 29483642 PMC5866759

[131] Wheeler EC, Vu AQ, Einstein JM et al. Pooled CRISPR screens with imaging on microraft arrays reveals stress granule-regulatory factors. Nat Methods. 2020; 17:636–42. 10.1038/s41592-020-0826-8. 32393832 PMC7357298

[132] Nitta N, Sugimura T, Isozaki A et al. Intelligent image-activated cell sorting. Cell. 2018; 175:266–76. 10.1016/j.cell.2018.08.028. 30166209

[133] Schraivogel D, Kuhn TM, Rauscher B et al. High-speed fluorescence image-enabled cell sorting. Science. 2022; 375:315–20. 10.1126/science.abj3013. 35050652 PMC7613231

[134] Kuhn TM, Paulsen M, Cuylen-Haering S Accessible high-speed image-activated cell sorting. Trends Cell Biol. 2024; 34:657–70. 10.1016/j.tcb.2024.04.007. 38789300

[135] Funk L, Su KC, Ly J et al. The phenotypic landscape of essential human genes. Cell. 2022; 185:4634–53. 10.1016/j.cell.2022.10.017. 36347254 PMC10482496

[136] Feldman D, Singh A, Schmid-Burgk JL et al. Optical pooled screens in human cells. Cell. 2019; 179:787–99. 10.1016/j.cell.2019.09.016. 31626775 PMC6886477

[137] Kolev NG, Franklin JB, Carmi S et al. The transcriptome of the human pathogen *Trypanosoma brucei* at single-nucleotide resolution. PLoS Pathog. 2010; 6:e1001090 10.1371/journal.ppat.1001090. 20838601 PMC2936537

[138] Vasquez JJ, Hon CC, Vanselow JT et al. Comparative ribosome profiling reveals extensive translational complexity in different *Trypanosoma brucei* life cycle stages. Nucleic Acids Res. 2014; 42:3623–37. 10.1093/nar/gkt1386. 24442674 PMC3973304

[139] Fiebig M, Kelly S, Gluenz E Comparative life cycle transcriptomics revises *L**eishmania mexican*a genome annotation and links a chromosome duplication with parasitism of vertebrates. PLoS Pathog. 2015; 11:e1005186 10.1371/journal.ppat.1005186. 26452044 PMC4599935

[140] Li Y, Shah-Simpson S, Okrah K et al. Transcriptome remodeling in *T**rypanosoma cruzi* and human cells during intracellular infection. PLoS Pathog. 2016; 12:e1005511 10.1371/journal.ppat.1005511. 27046031 PMC4821583

[141] Luzak V, Lopez-Escobar L, Siegel TN et al. Cell-to-Cell heterogeneity in trypanosomes. Annu Rev Microbiol. 2021; 75:107–28. 10.1146/annurev-micro-040821-012953. 34228491

[142] Hutchinson S, Foulon S, Crouzols A et al. The establishment of variant surface glycoprotein monoallelic expression revealed by single-cell RNA-seq of *Trypanosoma brucei* in the tsetse fly salivary glands. PLoS Pathog. 2021; 17:e1009904 10.1371/journal.ppat.1009904. 34543350 PMC8509897

[143] Briggs EM, Marques CA, Oldrieve GR et al. Profiling the bloodstream form and procyclic form *T**rypanosoma brucei* cell cycle using single-cell transcriptomics. eLife. 2023; 12:e86325 10.7554/eLife.86325. 37166108 PMC10212563

[144] Muller LSM, Cosentino RO, Forstner KU et al. Genome organization and DNA accessibility control antigenic variation in trypanosomes. Nature. 2018; 563:121–5. 10.1038/s41586-018-0619-8. 30333624 PMC6784898

[145] Howick VM, Peacock L, Kay C et al. Single-cell transcriptomics reveals expression profiles of *Trypanosoma brucei* sexual stages. PLoS Pathog. 2022; 18:e1010346 10.1371/journal.ppat.1010346. 35255094 PMC8939820

[146] Keneskhanova Z, McWilliam KR, Cosentino RO et al. Genomic determinants of antigen expression hierarchy in African trypanosomes. Nature. 2025; 642:182–90. 10.1038/s41586-025-08720-w. 40074895 PMC12137147

[147] Beaver AK, Keneskhanova Z, Cosentino RO et al. Tissue spaces are reservoirs of antigenic diversity for *Trypanosoma brucei*. Nature. 2024; 636:430–7. 10.1038/s41586-024-08151-z. 39478231 PMC11634766

[148] Faria JRC, Tinti M, Marques CA et al. An allele-selective inter-chromosomal protein bridge supports monogenic antigen expression in the African trypanosome. Nat Commun. 2023; 14:430–7. 10.1038/s41467-023-44043-y. 38081826 PMC10713589

[149] Vigneron A, O’Neill MB, Weiss BL et al. Single-cell RNA sequencing of *Trypanosoma brucei* from tsetse salivary glands unveils metacyclogenesis and identifies potential transmission blocking antigens. Proc Natl Acad Sci USA. 2020; 117:2613–21. 10.1073/pnas.1914423117. 31964820 PMC7007551

[150] Briggs EM, Rojas F, McCulloch R et al. Single-cell transcriptomic analysis of bloodstream *Trypanosoma brucei* reconstructs cell cycle progression and developmental quorum sensing. Nat Commun. 2021; 12:5268 10.1038/s41467-021-25607-2. 34489460 PMC8421343

[151] Briggs EM, Warren FSL, Matthews KR et al. Application of single-cell transcriptomics to kinetoplastid research. Parasitology. 2021; 148:1223–36. 10.1017/S003118202100041X. 33678213 PMC8311972

[152] Faria J, Glover L, Hutchinson S et al. Monoallelic expression and epigenetic inheritance sustained by a *Trypanosoma brucei* variant surface glycoprotein exclusion complex. Nat Commun. 2019; 10:3023 10.1038/s41467-019-10823-8. 31289266 PMC6617441

[153] Bard JE, Tylec BL, Dubey AP et al. Life stage-specific poly(A) site selection regulated by *Trypanosoma brucei* DRBD18. Proc Natl Acad Sci USA. 2024; 121:e2403188121 10.1073/pnas.2403188121. 38990950 PMC11260167

[154] Bishola Tshitenge T, Clayton C The *Trypanosoma brucei* RNA-binding protein DRBD18 ensures correct mRNA trans splicing and polyadenylation patterns. RNA. 2022; 28:1239–62. 10.1261/rna.079258.122. 35793904 PMC9380746

[155] Zappia MP, de Castro L, Ariss MM et al. A cell atlas of adult muscle precursors uncovers early events in fibre-type divergence in Drosophila. EMBO Rep. 2020; 21:e49555 10.15252/embr.201949555. 32815271 PMC7534622

[156] Datlinger P, Rendeiro AF, Boenke T et al. Ultra-high-throughput single-cell RNA sequencing and perturbation screening with combinatorial fluidic indexing. Nat Methods. 2021; 18:635–42. 10.1038/s41592-021-01153-z. 34059827 PMC7612019

[157] Lemaire LA, Cao C, Yoon PH et al. The hypothalamus predates the origin of vertebrates. Sci Adv. 2021; 7:eabf7452 10.1126/sciadv.abf7452. 33910896 PMC8081355

[158] Jun S, Lim H, Chun H et al. Single-cell analysis of a mutant library generated using CRISPR-guided deaminase in human melanoma cells. Commun Biol. 2020; 3:154 10.1038/s42003-020-0888-2. 32242071 PMC7118117

[159] Stadtmauer EA, Fraietta JA, Davis MM et al. CRISPR-engineered T cells in patients with refractory cancer. Science. 2020; 367:eaba7365 10.1126/science.aba7365. 32029687 PMC11249135

[160] Schofield JA, Hahn S Transcriptional noise, gene activation, and roles of SAGA and Mediator Tail measured using nucleotide recoding single-cell RNA-seq. Cell Rep. 2024; 43:114593 10.1016/j.celrep.2024.114593. 39102335 PMC11405135

[161] Zhou P, Shi H, Huang H et al. Single-cell CRISPR screens *in vivo* map T cell fate regulomes in cancer. Nature. 2023; 624:154–63. 10.1038/s41586-023-06733-x. 37968405 PMC10700132

[162] Joung J, Ma S, Tay T et al. A transcription factor atlas of directed differentiation. Cell. 2024; 187:3161 10.1016/j.cell.2024.04.038. 38697106

[163] Morris JA, Caragine C, Daniloski Z et al. Discovery of target genes and pathways at GWAS loci by pooled single-cell CRISPR screens. Science. 2023; 380:eadh7699 10.1126/science.adh7699. 37141313 PMC10518238

[164] Coelho MA, Strauss ME, Watterson A et al. Base editing screens define the genetic landscape of cancer drug resistance mechanisms. Nat Genet. 2024; 56:2479–92. 10.1038/s41588-024-01948-8. 39424923 PMC11549056

[165] Datlinger P, Rendeiro AF, Schmidl C et al. Pooled CRISPR screening with single-cell transcriptome readout. Nat Methods. 2017; 14:297–301. 10.1038/nmeth.4177. 28099430 PMC5334791

[166] Jaitin DA, Weiner A, Yofe I et al. Dissecting immune circuits by linking CRISPR-pooled screens with single-cell RNA-seq. Cell. 2016; 167:1883–96. 10.1016/j.cell.2016.11.039. 27984734

[167] Dixit A, Parnas O, Li B et al. Perturb-Seq: dissecting molecular circuits with scalable single-cell RNA profiling of pooled genetic screens. Cell. 2016; 167:1853–66. 10.1016/j.cell.2016.11.038. 27984732 PMC5181115

[168] Replogle JM, Norman TM, Xu A et al. Combinatorial single-cell CRISPR screens by direct guide RNA capture and targeted sequencing. Nat Biotechnol. 2020; 38:954–61. 10.1038/s41587-020-0470-y. 32231336 PMC7416462

[169] Xie S, Duan J, Li B et al. Multiplexed engineering and analysis of combinatorial enhancer activity in single cells. Mol Cell. 2017; 66:285–99. 10.1016/j.molcel.2017.03.007. 28416141

[170] Schraivogel D, Steinmetz LM, Parts L Pooled genome-scale CRISPR screens in single cells. Annu Rev Genet. 2023; 57:223–44. 10.1146/annurev-genet-072920-013842. 37562410

[171] Butterworth S, Kordova K, Chandrasekaran S et al. High-throughput identification of toxoplasma gondii effector proteins that target host cell transcription. Cell Host Microbe. 2023; 31:1748–62. 10.1016/j.chom.2023.09.003. 37827122 PMC12033024

[172] Sangare LO, Olafsson EB, Wang Y et al. *In vi**vo* CRISPR screen identifies TgWIP as a toxoplasma modulator of dendritic cell migration. Cell Host Microbe. 2019; 26:478–92. 10.1016/j.chom.2019.09.008. 31600500 PMC7060943

[173] Escrivani DO, Scheidt V, Tinti M et al. Competition among variants is predictable and contributes to the antigenic variation dynamics of African trypanosomes. PLoS Pathog. 2023; 19:e1011530 10.1371/journal.ppat.1011530. 37459347 PMC10374056

[174] Aitcheson N, Talbot S, Shapiro J et al. VSG switching in *Trypanosoma brucei*: antigenic variation analysed using RNAi in the absence of immune selection. Mol Microbiol. 2005; 57:1608–22. 10.1111/j.1365-2958.2005.04795.x. 16135228 PMC1618954

[175] Barbour AG, Dai Q, Restrepo BI et al. Pathogen escape from host immunity by a genome program for antigenic variation. Proc Natl Acad Sci USA. 2006; 103:18290–5. 10.1073/pnas.0605302103. 17101971 PMC1635980

[176] Boothroyd CE, Dreesen O, Leonova T et al. A yeast-endonuclease-generated DNA break induces antigenic switching in *Trypanosoma brucei*. Nature. 2009; 459:278–81. 10.1038/nature07982. 19369939 PMC2688456

[177] Glover L, Alsford S, Horn D DNA break site at fragile subtelomeres determines probability and mechanism of antigenic variation in African trypanosomes. PLoS Pathog. 2013; 9:e1003260 10.1371/journal.ppat.1003260. 23555264 PMC3610638

[178] Villiger L, Joung J, Koblan L et al. CRISPR technologies for genome, epigenome and transcriptome editing. Nat Rev Mol Cell Biol. 2024; 25:464–87. 10.1038/s41580-023-00697-6. 38308006

[179] Chen X, Du J, Yun S et al. Recent advances in CRISPR–Cas9-based genome insertion technologies. Mol Ther Nucleic Acids. 2024; 35:102138 10.1016/j.omtn.2024.102138. 38379727 PMC10878794

[180] Anzalone AV, Koblan LW, Liu DR Genome editing with CRISPR–Cas nucleases, base editors, transposases and prime editors. Nat Biotechnol. 2020; 38:824–44. 10.1038/s41587-020-0561-9. 32572269

[181] Lampe GD, King RT, Halpin-Healy TS et al. Targeted DNA integration in human cells without double-strand breaks using CRISPR-associated transposases. Nat Biotechnol. 2024; 42:87–98. 10.1038/s41587-023-01748-1. 36991112 PMC10620015

[182] Yarnall MTN, Ioannidi EI, Schmitt-Ulms C et al. Drag-and-drop genome insertion of large sequences without double-strand DNA cleavage using CRISPR-directed integrases. Nat Biotechnol. 2023; 41:500–12. 10.1038/s41587-022-01527-4. 36424489 PMC10257351

[183] Durrant MG, Perry NT, Pai JJ et al. Bridge RNAs direct programmable recombination of target and donor DNA. Nature. 2024; 630:984–93. 10.1038/s41586-024-07552-4. 38926615 PMC11208160

[184] Saito M, Xu P, Faure G et al. Fanzor is a eukaryotic programmable RNA-guided endonuclease. Nature. 2023; 620:660–8. 10.1038/s41586-023-06356-2. 37380027 PMC10432273

[185] Gu J, Iyer A, Wesley B et al. Mapping multimodal phenotypes to perturbations in cells and tissue with CRISPRmap. Nat Biotechnol. 2024; 10.1038/s41587-024-02386-x. PMC12263428 39375448

[186] Morris JA, Sun JS, Sanjana NE Next-generation forward genetic screens: uniting high-throughput perturbations with single-cell analysis. Trends Genet. 2024; 40:118–33. 10.1016/j.tig.2023.10.012. 37989654 PMC10872607

[187] Alvarez-Jarreta J, Amos B, Aurrecoechea C et al. VEuPathDB: the eukaryotic pathogen, vector and host bioinformatics resource center in 2023. Nucleic Acids Res. 2024; 52:D808–16. 10.1093/nar/gkad1003. 37953350 PMC10767879

[188] Amos B, Aurrecoechea C, Barba M et al. VEuPathDB: the eukaryotic pathogen, vector and host bioinformatics resource center. Nucleic Acids Res. 2022; 50:D898–911. 10.1093/nar/gkab929. 34718728 PMC8728164

[189] Le Mercier P, Bolleman J, de Castro E et al. SwissBioPics-an interactive library of cell images for the visualization of subcellular location data. Database. 2022; 2022:baac026 10.1093/database/baac026. 35411389 PMC9216577

